# Novel Thiazole Derivatives Containing Imidazole and Furan Scaffold: Design, Synthesis, Molecular Docking, Antibacterial, and Antioxidant Evaluation

**DOI:** 10.3390/molecules29071491

**Published:** 2024-03-27

**Authors:** Fatimah Agili

**Affiliations:** Department of Physical Sciences, Chemistry Division, College of Science, Jazan University, P.O. Box 114, Jazan 45142, Saudi Arabia; fagili@jazanu.edu.sa

**Keywords:** thiazol, imidazole, furan, molecular docking, DNA gyrase B, antibacterial, antioxidant

## Abstract

Carbothioamides **3a**,**b** were generated in high yield by reacting furan imidazolyl ketone **1** with *N*-arylthiosemicarbazide in EtOH with a catalytic amount of conc. HCl. The reaction of carbothioamides **3a**,**b** with hydrazonyl chlorides **4a**–**c** in EtOH with triethylamine at reflux produced 1,3-thiazole derivatives **6a**–**f**. In a different approach, the 1,3-thiazole derivatives **6b** and **6e** were produced by reacting **3a** and **3b** with chloroacetone to afford **8a** and **8b**, respectively, followed by diazotization with 4-methylbenzenediazonium chloride. The thiourea derivatives **3a** and **3b** then reacted with ethyl chloroacetate in ethanol with AcONa at reflux to give the thiazolidinone derivatives **10a** and **10b**. The produced compounds were tested for antioxidant and antibacterial properties. Using phosphomolybdate, promising thiazoles **3a** and **6a** showed the best antioxidant activities at 1962.48 and 2007.67 µgAAE/g dry samples, respectively. Thiazoles **3a** and **8a** had the highest antibacterial activity against *S. aureus* and *E. coli* with 28, 25 and 27, 28 mm, respectively. Thiazoles **3a** and **6d** had the best activity against *C. albicans* with 26 mm and 37 mm, respectively. Thiazole **6c** had the highest activity against *A. niger*, surpassing cyclohexamide. Most compounds demonstrated lower MIC values than neomycin against *E. coli*, *S. aureus* and *C. albicans*. A molecular docking study examined how antimicrobial compounds interact with DNA gyrase B crystal structures. The study found that all of the compounds had good binding energy to the enzymes and reacted similarly to the native inhibitor with the target DNA gyrase B enzymes’ key amino acids.

## 1. Introduction

Currently, there is an increasing occurrence of public health problems resulting from antimicrobial resistance (AMR) due to the use of antibiotics. Hence, it is crucial to identify an innovative pharmaceutical intervention that efficiently tackles the obstacles presented by antimicrobial resistance (AMR) [1]. Pharmaceuticals incorporating heterocyclic nuclei have demonstrated notable chemotherapeutic effectiveness and have contributed to the development of novel therapeutic interventions [2]. In contemporary clinical practice, a variety of heterocyclic compounds are presently employed for the therapeutic management of infectious diseases. Medicines containing heterocyclic rings are of great significance [3].

Imidazole exhibits amphoteric properties due to the presence of two distinct types of lone pairs (delocalized and non-delocalized). The dissociation constants (pKa) of the two nitrogen atoms are 7 and 14.9, respectively. The amphoteric imidazole ring is susceptible to both electrophilic and nucleophilic attacks [4]. Imidazole exhibits lower acidity compared to phenol, imides, and carboxylic acids, except for alcohols, due to its pKa value of 14.9. An imidazole with a pKa of approximately 7 exhibits a basicity that is 60 times greater than that of pyridine. The nitrogen atom located at the beginning of the imidazole ring possesses an acidic proton [5].

Azoles such as imidazole and thiazole are extremely valuable molecules in the domains of organic synthesis and biological activity. As long as 166 years ago, researchers synthesized imidazoles with modest efficiency by reacting glyoxal, formaldehyde, and ammonia [6]. The synthesis of trisubstituted imidazoles was reported in 1882 by a combination of *α*-diketones, aldehydes, and two equivalents of ammonia. In 1935, Weidenhagen demonstrated a high-yield manufacture of imidazoles by mixing α-hydroxy ketones, ammonia, aldehydes, hydrogen sulfide, and cupric acetate. A large yield of tetrasubstituted imidazoles was produced by replacing one equivalent of ammonia with an amine [7]. Imidazole derivatives exhibit intriguing chemical properties and serve as flexible components in various naturally occurring molecules [8]. They possess a diverse array of uses in the fields of pharmacology and industry [9,10,11,12]. Imidazoles possess a wide range of biological characteristics, such as their ability to inhibit cancer growth, reduce inflammation, combat microbial infections, and lower blood pressure. Ionic liquids with imidazole salt structures have also been very important as electrolytes and solvents that are safe for the environment in chemical synthesis, as stable ligands in metalloenzymes, and as liquid crystals [13,14,15,16,17].

**Figure 1:**
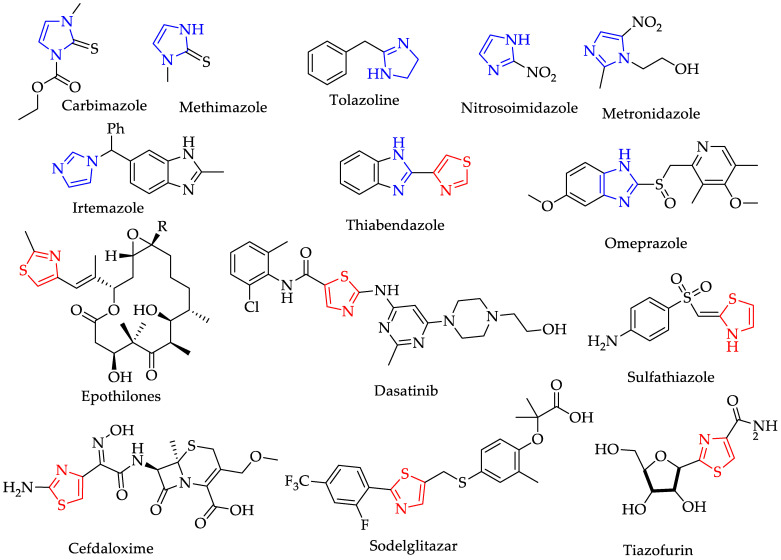
Some drugs containing imidazole and thiazole components.

Conversely, the thiazole component is found in many natural substances [18,19], and its derivatives have a wide range of uses in the development of drugs for treating various diseases [20,21,22]. They demonstrated remarkable pharmacological properties, such as antifungal [23,24,25], antibacterial [26,27,28], anti-inflammatory [29,30], analgesic [31], anti-cancer [32,33], and anticonvulsant activity [34,35]. A recent paper has reported that thiazole derivatives have strong antibacterial activity [36,37]. Moreover, there are numerous pharmaceuticals that contain imidazole and thiazole components (Figure 1).

Given the multitude of advantages and as a logical extension of my efforts [38,39,40,41,42,43], in this study, I describe the creation of new thiazole compounds that have a 2-(furan-2-yl)-1*H*-imidazole component, which acts as an antibacterial agent.

## 2. Results

### 2.1. Chemistry

**Scheme 1:**
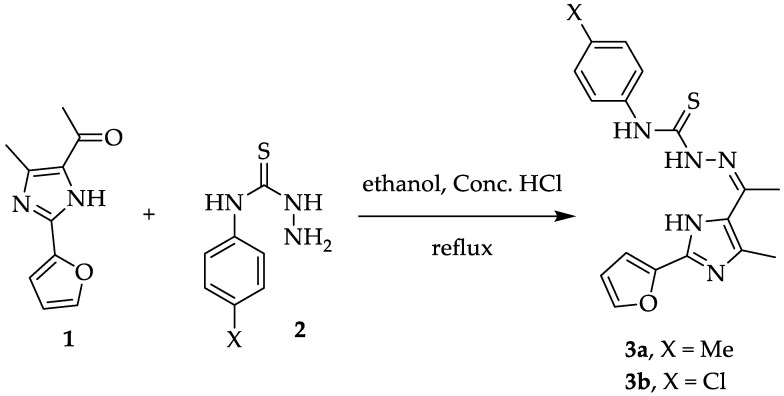
Synthesis of 2-(1-(2-(furan-2-yl)-4-methyl-1H-imidazol-5-yl)ethylidene)-N-arylhydrazine-1-carbothioamide **3a**,**b**.

Carbothioamide **3a**,**b** was produced by reacting 1-(2-(furan-2-yl)-4-methyl-1*H*-imidazol-5-yl)ethan-1-one **1** [44] with *N*-arylhydrazinecarbothioamide [45] in absolute EtOH containing five drops of concentrated HCl (Scheme 1). The structure of thiosemicarbazones **3a** and **3b** was identified based on elemental analyses and spectral data. The IR spectrum of **3a** and **3b** exhibited distinct absorption bands resulting from two NH and C=S functional groups at 3254–3207 and 1269–1265 cm^−1^, respectively, but lacking a carbonyl group [37,46]. The ^1^H NMR spectra showed two novel D_2_O exchangeable singlet signals at δ 9.15–8.90 and 10.15–10.12 ppm; in addition, NH-imidazole appeared at δ 13.65–13.55 ppm. Furthermore, there are two distinct doublet signals from the aromatic protons, with chemical shifts at δ 7.30–7.90 ppm. In addition, three protons of furan appeared as doubles of doubles and two doublet signals. The ^13^C NMR spectrum displayed a resonance peak at 176.48–184.14 ppm corresponding to the C=S functional group (Figures S1–S4, Supplementary Data).

**Scheme 2:**
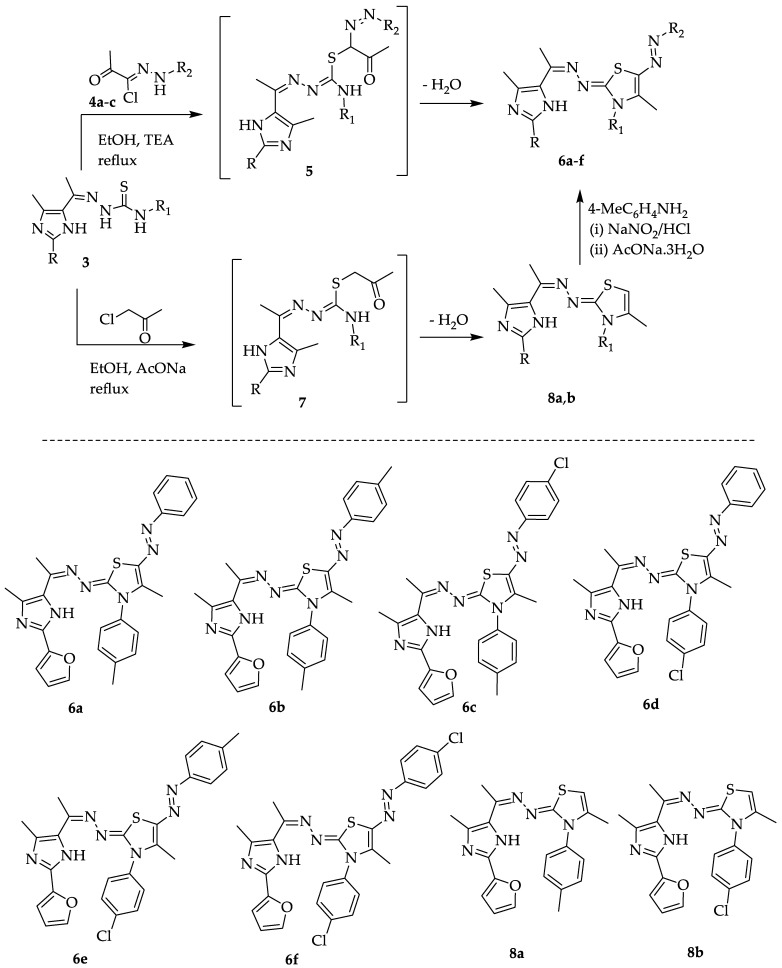
Synthesis of thiazole derivatives **6a**–**f** and **8a**,**b**.

**Scheme 3:**
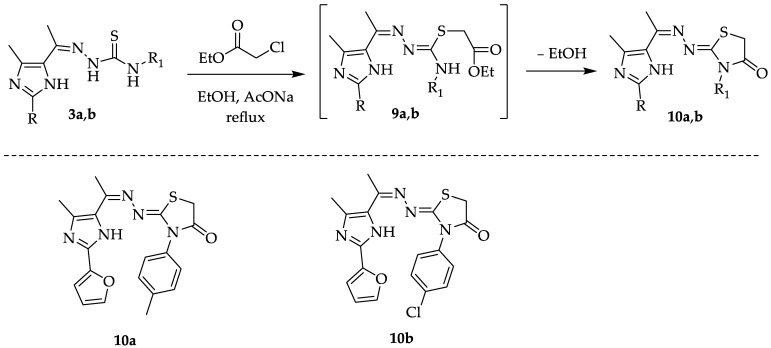
Synthesis of thiazolidinone derivatives **10a**,**b**.

The reactivity of the thiourea group was evaluated by subjecting carbothioamides **3a** and **3b** to various reagents, as illustrated in Scheme 2 and Scheme 3. The reaction of compounds **3a** and **3b** with hydrazonyl chloride **4a**–**c** in absolute EtOH with five drops of triethylamine at reflux temperature results in the formation of 1,3-thiazole derivatives **6a**–**f**. This reaction proceeds through nucleophilic substitution followed by cyclization and gives good yields. Alternatively, 1,3-thiazole derivatives **6b** and **6e** were synthesized using a different method. This involved reacting **3a**,**b** with chloroacetone to produce **8a**,**b** in a high yield. The next step in the process was diazotization, which was achieved by employing 4-methylbenzenediazonium chloride (Scheme 2).

The structure of compounds **6a**–**f** and **8a**,**b** was verified based on elemental analyses and spectral data. The IR spectrum of compound **6b**, as an illustrative instance, showed the absence of NH and C=S peaks at 3251 and 1269 cm^−1^. The ^1^H-NMR spectra of **6b** exhibited two new singlet signals at δ 2.17 and 2.41 ppm, which were allocated to two methyl groups. Furthermore, increasing aromatic signals at δ 7.38–7.48 ppm (multiplet) and 7.73–7.76 ppm (doublet of doublet, *J* = 6, 3 Hz) were seen, which can be attributed to 4-methylbenzene. The ^13^C-NMR spectra indicated the absence of a C=S signal at 176.48 ppm and the presence of 24 carbon signals (Figures S5–S20, Supplementary Data). In addition, the mass spectra of **6b** exhibited a peak at m/z 509, corresponding to the [M^+^, 35%] ion. These findings demonstrate that the thioamide group played a crucial role in the cyclization reaction with hydrazonyl chlorides, resulting in the formation of 1,3-thiazole derivatives **6a**–**f**.

Compounds **3a** and **3b** underwent a reaction with ethyl chloroacetate in ethanol, in the presence of AcONa under reflux conditions. This reaction resulted in the formation of thiazolidinone derivatives **10a** and **10b**, respectively, with high yields (Scheme 3). The IR spectra of **10b** displayed a prominent new absorption band at 1743 cm^−1^, which corresponds to the presence of a C=O group. Additionally, the thioamide moiety was no longer detectable. The ^1^H NMR spectra showed a novel singlet for the methylene group at δ 4.05 ppm. The ^13^C NMR spectra of **10b** displayed 17 carbon peaks, such as CH_2_ and CO, located at δ 35.10 and 173.70 ppm, respectively, within the thiazole ring (Figures S21–S24, Supplementary Data). The mass spectrum exhibited a prominent peak at 413 (90%) for the [M^+^] ion.

### 2.2. Antioxidant Activity

The antioxidant activity of the produced compounds that were created through the use of the phosphomolybdate technique is depicted in Table 1. Based on the results, it was observed that compounds **6a**, **3a**, **8b**, **6d**, **6b**, **6e**, **6c**, and **6f** exhibited significant overall antioxidant activity. The compounds exhibited a range of values spanning from 2007.67 to 1654.76 µg AAE/g dry sample. The greatest activity was observed in thiazole compounds that incorporated an aryldiazenyl function. Furthermore, it was observed that compounds **3b**, **8a**, **10a**, and **10b** exhibited a reduced antioxidant capacity in comparison to the compounds described earlier. The values for these compounds were 1369.14, 1321.74, 1396.06, and 1370.87 µg AAE/g dry sample, respectively.

### 2.3. Antimicrobial Activity and MIC Concentrations

The antimicrobial activity of the produced compounds was evaluated by testing them against various types of microbial strains. These strains included the filamentous fungus (*A. niger*), the G+ve bacterium (*S. aureus*), the G-ve bacterium (*E. coli*), and yeast (*C. albicans*). According to the findings presented in Table 2, compounds **3a**, **3b**, **8a**, **8b**, **6d**, **6e**, **6f**, **10a**, and **10b** demonstrated stronger activity against *S. aureus*. The inhibition values for these compounds were 28, 23, 25, 24, 23, 21, 20, 22, and 21 mm, respectively. Compounds **6a**, **6b**, and **6c** exhibited no actions against *S. aureus*. The findings on the impact of these compounds on *E. coli* revealed that compounds **3a** (27 mm), **8a** (28 mm), **8b** (25 mm), **6d** (21mm), **10a** (25 mm), and **10b** (26 mm) exhibited potential activators against the bacteria. In contrast, compounds **3b** (18 mm), **6e** (16 mm), and **6f** (18 mm) exhibited activity that was below the threshold. Additionally, compounds **6a**, **6b**, and **6c** did not exhibit any significant activity against *E. coli*. When the compounds were evaluated against *C. albicans*, it was discovered that compounds **3a**, **8a**, **8b**, **6d**, **6f**, **10a**, and **10b** exhibited substantial activity. The inhibition values for these compounds were 26, 21, 23, 26, 21, 26, and 24 mm, respectively. Furthermore, compounds **3b** and **6e** exhibited moderate activity (17 and 19 mm, respectively), but compounds **6a**, **6b**, and **6c** exhibited detrimental effects on *C. albicans*. When *A. niger*, a filamentous fungus, was employed as the fungal test strain, the results suggested that compounds **6c** and **6f** had the potential to inhibit the growth of fungi (37 and 25 mm, respectively). Lower activity was seen in compounds **3b**, **6d**, and **6e**, which were 16 mm, 15 mm, and 16 mm, respectively. The remaining compounds were not capable of functioning as antifungal agents.

The minimum inhibitory concentration (MIC) values of these compounds were determined using the visual method outlined by Sarker et al. [47] with resazurin dye as an indicator. Table 3 showed that compounds **3a**, **3b**, **8a**, **8b**, **6d**, **6e**, **6f**, **10a** and **10b** demonstrated lower MIC values than neomycin against *E. coli.* The values ranged from 4.88 to 39.06 µg/mL. Compounds **3a**, **3b**, **8a**, and **10a** showed significant MIC values of 4.88, 19.53, 9.77, and 19.53 µg/mL, respectively, against *S. aureus*. In addition, MIC values for compounds **3a**, **3b**, **6d**, **6e**, **6f**, **8b**, and **10b** against yeast *C. albicans* were 156.25 µg/mL (Table 3).

### 2.4. Molecular Docking Results

Prompted by the broad antimicrobial activity results, compounds **3a**, **3b**, **8a**, **8b**, and **10b** were designated for molecular docking against DNA gyrase B (the principal site of action for Gram-positive as well as Gram-negative bacteria) [48]. DNA gyrase plays an indispensable role in bacterial replication and compaction [49]. Inhibition of DNA gyrase prevents the relaxation of supercoiled DNA required for replication, consequently preventing cell division and ultimately leading to bacterial death [50,51].

Consequently, the X-ray crystallographic structure of *S. aureus* DNA gyrase B (PDB: 3U2D) with the native ligand (08B) and the X-ray crystallographic structure of *E. coli* DNA gyrase B (PDB: 1S14) with the native ligand (NOV) were downloaded from PDB https://www.rcsb.org (accessed on 27 February 2024). Firstly, the co-crystallized ligands (08B) and (NOV) were re-landed in the enzyme’s active cave to ensure the technique of molecular docking. It was noticed that the re-loaded native ligands revealed a docking score (S) of −8.0 and −8.3 kcal/mol, respectively, and reproduced all interactions with the key amino acids of the active cave. For *S. aureus* DNA gyrase B (PDB: 3U2D), as previously reported for docking 08B, it was stabilized within the active site of DNA gyrase B through hydrogen bonds to the basic amino acids ASP81, ARG144, and THR173 [52] (Figure S25, Supplementary Data). But *E. coli* DNA gyrase B (PDB: 1S14) has been linked to Asp1077, ARG1072, ASN1042, THR1163, and ASP1069 via hydrogen bond interaction and further alkyl and π–alkyl interactions [53] (Figure S26, Supplementary Data). The mode with the lowest binding energy and 0 Å RMSD (root mean square deviation) has been considered adequate and most complex with the receptor for investigation.

### 2.5. Assessment of the Binding Interaction of Studied Compounds with S. aureus DNA Gyrase B (PDB: 3U2D)

Table 4 shows the docking results of the studied compounds. All compounds had excellent binding energy for the active site of *S. aureus* DNA gyrase B (PDB: 3U2D) by values in the order of **3a** (−8.4 Kcal/mol) > **10b** (−8.3 Kcal/mol) > **8a**, **8b** (−8.1 Kcal/mol) > **3b** (−7.9 Kcal/mol) against the native ligand (**08B**) of −8.0 Kcal/mol.

**Figure 2:**
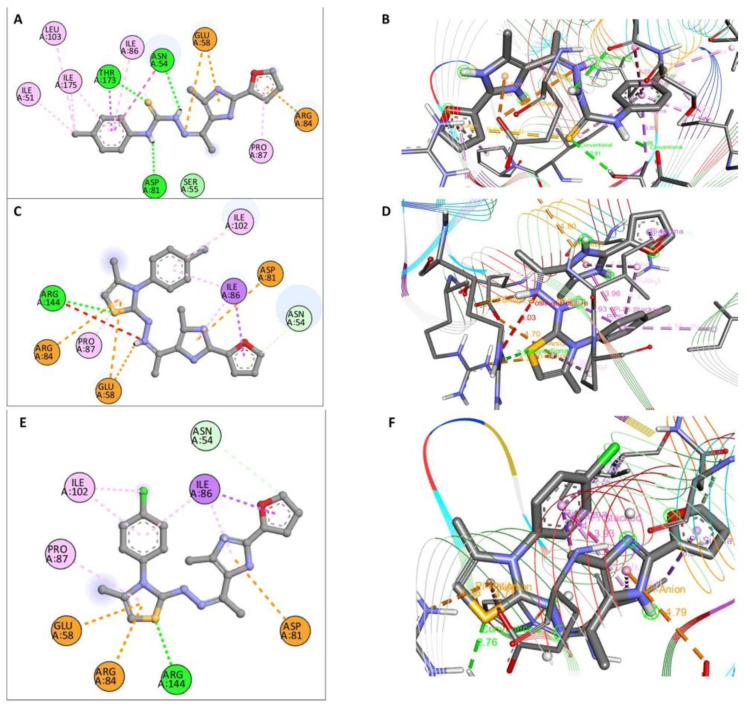
(**A**,**C**,**E**): The 2D representation of the active compounds **3a**, **8a**, and **8b** inside the active cave of *S. aureus* DNA gyrase B (PDB: 3U2D). (**B**,**D**,**F**): The 3D configuration of the active compounds **3a**, **8a**, and **8b** inside the active cave of *S. aureus* DNA gyrase B (PDB: 3U2D).

Although all the tested compounds have been slid into the same position as the ligand, only three compounds (**3a**, **8a**, and **8b**) interacted with the key amino acids THR173, ARG144, and ASP81 of the active pocket of *S. aureus* DNA gyrase B (PDB: 3U2D). Compound **3a** demonstrated three conventional hydrogen bonds through (C=S), NH with the side chain residues THR173, ARG144, and ASP81 of the active pocket of *S. aureus* DNA gyrase B (PDB: 3U2D) (Figure 2A,B). Compound **8a** showed one hydrogen bond with the key amino acid ARG144 and was linked with the key amino acid (ASP81) via pi–cation interaction (Figure 2C,D). Also, compound **8b** exhibited a hydrogen bond with the key amino acid ARG144 besides pi–cation interaction with ASP81 (Figure 2E,F) (Table 5). 

### 2.6. Assessment of the Interaction Mode of the Studied Compounds with E. coli DNA Gyrase B (PDB: 1S14)

**Figure 3:**
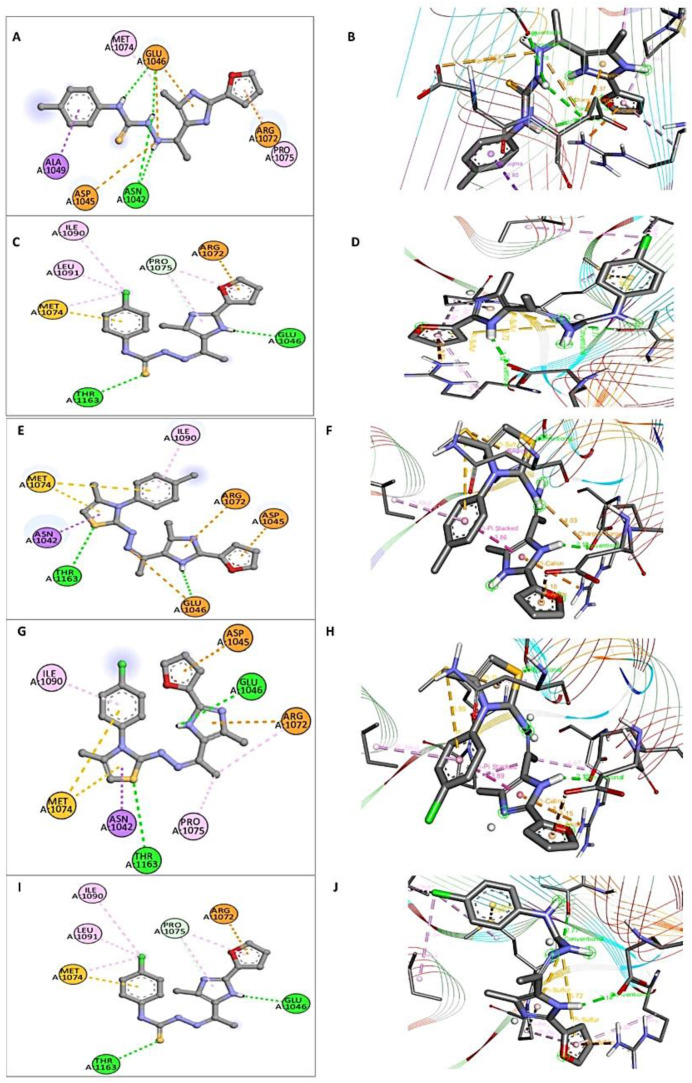
(**A**,**C**,**E**,**G**,**I**): The 2D representation of the active compounds **3a**, **3b**, **8a**, **8b**, and **10b** inside the active cave of *E. coli* DNA gyrase B (PDB: 1S14). (**B**,**D**,**F**,**H**,**J**): The 3D configuration of the active compounds **3a**, **3b**, **8a**, **8b**, and **10b** inside the active cave of *E. coli* DNA gyrase B (PDB: 1S14).

The studied compounds confirmed excellent binding energy for the active site of *E. coli* DNA gyrase B (PDB: 1S14) ranging from −6.5 to −6.9 Kcal/mol against the native ligand (NOV) of −8.3 Kcal/mol in the order of **3b** (−6.9 Kcal/mol) > **3a** (−6.8 Kcal/mol) > **8a** (−6.6 Kcal/mol) > **8b and 10b** (−6.5 Kcal/mol). All compounds under test demonstrated good binding interaction inside the active cave of the *E. coli* DNA gyrase B (PDB: 1S14) (Table 4). They demonstrated that good binding interactions with the active cave of the *E. coli* DNA gyrase B (PDB: 1S14) form multiple interactions with the key amino acids of *E. coli* DNA gyrase B similar to NOV (Figure 3). Compound **3a** recreated the contact with the key amino acid ASN1042 and linked with the key amino acid ARG1072 via pi–cation interaction. Compounds **3b**, **8a**, **8b**, and **10b** exhibited one hydrogen bond with THR1163 (the key amino acid) via π–cation interactions with ARG1072 (Figure 3) (Table 5). 

## 3. Experimental Section

### 3.1. General Information

Melting points were uncorrected and measured using a digital Gallen-Kamp MFB-595 device. Using KBr pellets, IR spectra were acquired using a Shimadzu FTIR 440 spectrometer (Shimadzu, Kyoto, Japan). The data were analyzed with an MS-50 Kratos (A.E.I.) spectrometer, which was used to obtain mass spectra at 70 eV. Using CDCl_3_ or DMSO-d_6_ as an internal standard and TMS as an external standard, ^1^H NMR (300 MHz) and ^13^C NMR (75 MHz) spectra were acquired on a Bruker model UltraShield NMR spectrometer. The units used to report chemical changes are δ ppm. Thin layer chromatography (TLC) was used to assess the homogeneity of the products and the course of the reactions. 

### 3.2. Synthesis

#### 3.2.1. Synthesis of Thiosemecarbazone Derivatives **3a** and **3b**

A solution of 1-(2-(furan-2-yl)-4-methyl-1*H*-imidazol-5-yl)ethan-1-one **1** (7.6 g, 40 mmol), *N*-(*p*-tolyl)hydrazinecarbothioamide **2a** (40 mmol, 0.73 g) or *N*-(4-chlorophenyl)hydrazinecarbothioamide **2b** (40 mmol, 0.81 g) in absolute ethanol (25 mL) containing conc. HCl (0.5 mL) was heated under reflux for 2–3 h (TLC). The resulting mixture was then cooled to room temperature, and the crystals that formed were separated by filtration to obtain thiosemicarbazone derivatives **3a** and **3b**.

*2-(1-(2-(furan-2-yl)-4-methyl-1H-imidazol-5-yl)ethylidene)-N-(p-tolyl)hydrazine-1-carbothioamide* (**3a**). Color: yellow crystals (EtOH); yield (90%); m.p. 199–200 °C. IR (KBr, υ_max_, cm^−1^): 3331, 3251, 3210 (3NH), 1546 (C=N), 1269 (C=S); ^1^H NMR (300 MHz, DMSO-*d_6_*) δ (ppm): 2.29 (s, Ph–CH_3_), 2.58 (s, N=C–CH_3_), 2.67 (s, CH_3_–imidazole), 6.64–6.71 (dd, *J* = 12, 3 Hz, 1H–furan), 7.13–7.14 (d, *J* = 3 Hz, 1H–furan), 7.30–7.40 (2d, *J* = 9, 6 Hz, 2H–Ar), 7.53–7.63 (2d, *J* = 9, 6 Hz, 2H-Ar), 7.86–7.89 (d, *J* = 9 Hz, 1H–furan), 9.15 (s, NH, D_2_O exchangeable), 10.15 (s, NH, D_2_O exchangeable), 13.55 (s, NH–imidazole, D_2_O exchangeable); ^13^C NMR (DMSO-*d_6_*) δc (ppm): 14.81, 18.18, 21.42, 91.29, 112.38, 117.46, 120.54, 123.28, 129.12, 134.59, 138.37, 145.75, 147.03, 150.37, 160.21, 176.48; EI-MS 353 [M^+^, 30%]; Anal. Calcd. for C_18_H_19_N_5_OS: C, 61.17; H, 5.42; N, 19.82. Found: C, 60.98; H, 5.38; N, 19.76.

*N-(4-chlorophenyl)-2-(1-(2-(furan-2-yl)-4-methyl-1H-imidazol-5-yl)ethylidene)hydrazine-1-carbothioamide* (**3b**). Color: white crystals (EtOH); yield (87%); m.p. 210–212 °C. IR (KBr, υ_max_, cm^−1^): 3325, 3254, 3207 (3NH), 1535 (C=N), 1265 (C=S); ^1^HNMR (300 MHz, DMSO-*d_6_*) δ (ppm): 2.66 (s, N=C–CH_3_), 2.72 (s, imidazole–CH_3_), 7.11–7.15 (dd, *J* = 9, 3 Hz, 1H–furan), 7.33–7.35 (d, *J* = 6 Hz, 1H-furan), 7.46–7.55 (2d, *J* = 9, 6 Hz, 2H–Ar), 7.80–7.90 (2d, *J* = 9, 6 Hz, 2H–Ar), 8.11–8.12 (d, *J* = 3 Hz, 1H–furan), 8.90 (s, NH, D_2_O exchangeable), 10.12 (s, NH, D_2_O exchangeable), 13.65 (s, NH–imidazole, D_2_O exchangeable); ^13^C NMR (DMSO-*d_6_*) δc (ppm): 14.82, 21.42, 105.42, 112.38, 117.46, 126.07, 128.40, 129.12, 134.59, 138.37, 145.75, 147.03, 150.37, 160.21, 184.14 (C=S); EI-MS 373 [M^+^, 27%]; Anal. Calcd. for C_17_H_16_ClN_5_OS: C, 54.62; H, 4.31; N, 18.73. Found: C, 54.46; H, 4.25; N, 1852.

#### 3.2.2. Synthesis of 1,3-Thiazole Derivatives **6a**–**f**

Method A: **Equal** amounts of carbothioamide derivatives **3a** or **3b** (1 mmol, 0.353 or 0.374 g) and hydrazonoyl chloride **4a–c** (1 mmol, 0.197 g for **4a** or 0.211 g for **4b** or 0.231 g for **4c**) were combined in absolute ethanol (25 mL). A few drops of triethylamine were added, and the mixture was heated under reflux for 2–3 h. The resulting solid products were filtered off, washed with ethanol, and recrystallized from EtOH to obtain **6a–f**.

Method B: Compound **8a** or **8b** (2 mmol, 0.783 for **8a** or 0.824 g for **8b**) was dissolved in ethanol (30 mL) and agitated. Then, sodium acetate trihydrate (0.26 g, 2 mmol) was added to the solution. After agitating for 15 min, the mixture was cooled to 0 °C and subjected to a cold solution of *p*-toluidine (2 mmol, 0.214 g) in conc. HCl (1.5 mL) along with a solution of sodium nitrite (0.14 g, 2 mmol) in water (3 mL). The solution was agitated for an additional 2 h at a temperature range of 0–5 °C and subsequently stored for 8 h in a refrigerator at 4 °C. The solid obtained was recovered via filtration, then extensively rinsed with water and subsequently dried. The raw product underwent crystallization from ethanol, resulting in the formation of compounds **6b** and **6e**, respectively.

*2-((-1-(2-(furan-2-yl)-4-methyl-1H-imidazol-5-yl)ethylidene)hydrazineylidene)-4-methyl-5-(phenyldiazenyl)-3-(p-tolyl)-2,3-dihydrothiazole* (**6a**). Color: Brownish red crystal (EtOH); yield (85%); m.p. 124–125 °C. IR (KBr, υ_max_, cm^−1^): 3340 (NH), 1592 (C=N); ^1^HNMR (300 MHz, DMSO-*d_6_*) δ (ppm): 2.18 (s, thiazole–CH_3_), 2.37 (s, Ph–CH_3_), 2.45 (s, N=C–CH_3_), 2.58 (s, imidazole–CH_3_), 6.83–6.87 (dd, *J* = 6, 3 Hz, 1H–furan), 7.08–7.10 (d, *J* = 6 Hz, 1H–furan), 7.38–7.51 (m, 5H–Ar), 7.58–7.61 (dd, *J* = 6, 3 Hz, 2H–Ar), 7.71–7.75 (dd, *J* = 6, 3 Hz, 2H–Ar), 7.87–7.88 (d, *J* = 3 Hz, 1H–furan), 13.17 (s, NH–imidazole, D_2_O exchangeable); ^13^C NMR (DMSO-*d_6_*) δc (ppm): 11.12, 12.57, 14.82, 21.42, 96.26, 110.25, 115.74, 120.54, 126.07, 129.12, 130.74, 131.66, 132.56, 135.18, 136.38, 141.69, 142.40, 144.45, 151.17, 154.18, 157.30, 162.47, 167.51; EI-MS 495 [M^+^]; Anal. Calcd. for C_27_H_25_N_7_OS: C, 65.43; H, 5.08; N, 19.78. Found: C, 65.27; H, 5.00; N, 19.56.

*2-((-1-(2-(furan-2-yl)-4-methyl-1H-imidazol-5-yl)ethylidene)hydrazineylidene)-4-methyl-3-(p-tolyl)-5-(p-tolyldiazenyl)-2,3-dihydrothiazole* (**6b**). Color: Brownish red crystals (EtOH); yield (83%); m.p. 125–126 °C. IR (KBr, υ_max_, cm^−1^): 3343 (NH), 1600 (C=N); ^1^HNMR (300 MHz, DMSO-*d_6_*) δ (ppm): 2.17 (s, 3H, thiazole–CH_3_, 2.41 (s, Ph–CH_3_), 2.42 (s, Ph–CH_3_), 2.54 (s, N=C–CH_3_), 2.58 (s, 3H, imidazole–CH_3_), 6.59–6.61 (t, 1H–furan), 7.08–7.11 (d, *J* = 9 Hz, 1H–furan), 7.38–7.48 (m, 6H–Ar), 7.73–7.76 (dd, *J* = 6, 3 Hz, 2H–Ar), 8.08–8.11 (d, *J* = 9 Hz, 1H–furan), 12.74 (s, 1H, NH–imidazole, D_2_O exchangeable); ^13^C NMR (DMSO-*d_6_*) δc (ppm): 12.34, 13.10, 15.22, 20.84, 21.92, 106.69, 111.85, 116.85, 121.61, 126.29, 129.29, 130.75, 132.92, 136.33, 137.23, 138.87, 141.27, 142.78, 145.68, 151.18, 152.37, 157.72, 162.63, 168.15; EI-MS 509 [M^+^, 35%]; Anal. Calcd. for C_28_H_27_N_7_OS: C, 65.99; H, 5.34; N, 19.24. Found: C, 65.78; H, 5.23; N, 19.03.

*5-((4-chlorophenyl)diazenyl)-2-((1-(2-(furan-2-yl)-4-methyl-1H-imidazol-5-yl)ethylidene)hydrazineylidene)-4-methyl-3-(p-tolyl)-2,3-dihydrothiazole* (**6c**). Color: Brownish red crystals (EtOH); yield (82%); m.p. 110–112 °C. IR (KBr, υ_max_, cm^−1^): 3347 (NH), 1604 (C=N); ^1^HNMR (300 MHz, DMSO-*d_6_*) δ (ppm): 2.18 (s, 3H, thiazole–CH_3_), 2.24 (s, 3H, Ph–CH_3_), 2.42 (s, N=C–CH_3_), 2.58 (s, imidazole–CH_3_), 6.72–6.75 (dd, *J* = 6, 3 Hz, 1H–furan), 7.16–7.19 (d, *J* = 9 Hz, 1H–furan), 7.42–7.50 (m, 6H–Ar), 7.78–7.84 (2d, *J* = 6, 3 Hz, 2H–Ar), 8.12–8.15 (d, *J* = 9 Hz, 1H–furan), 13.45 (s, 1H, NH–imidazole, D_2_O exchangeable); ^13^C NMR (DMSO-*d_6_*) δc (ppm): 11.12, 14.82, 16.90, 21.42, 105.42, 112.38, 115.76, 125.01, 126.07, 126.52, 129.12, 132.01, 136.20, 137.22, 139.68, 141.24, 142.54, 144.74, 151.87, 154.49, 157.83, 162.41, 169.38; EI-MS 530 [M^+^]; Anal. Calcd. for C_27_H_24_ClN_7_OS: C, 61.18; H, 4.56; N, 18.50; O, 3.02; S, 6.05; Found: C, 61.03; H, 4.42; N, 18.38; O, 2.91; S, 5.86.

*3-(4-chlorophenyl)-2-((1-(2-(furan-2-yl)-4-methyl-1H-imidazol-5-yl)ethylidene)hydrazineylidene)-4-methyl-5-(phenyldiazenyl)-2,3-dihydrothiazole* (**6d**). Color: Brownish red crystals (EtOH); yield (86%); m.p. 113–114 °C. IR (KBr, υ_max_, cm^−1^): 3340 (NH), 1585 (C=N); ^1^HNMR (300 MHz, DMSO-*d_6_*) δ (ppm): 2.18 (s, 3H, thiazole–CH_3_), 2.55 (s, N=C–CH_3_), 2.58 (s, imidazole–CH_3_), 6.83–6.87 (dd, *J* = 6, 3 Hz, 1H–furan), 7.29–7.30 (d, *J* = 3 Hz, 1H–furan), 7.41–7.49 (m, 7H–Ar), 7.72–7.76 (dd, *J* = 9, 6 Hz, 2H–Ar), 8.08–8.11 (d, *J* = 9 Hz, 1H–furan), 13.17 (s, 1H, NH-imidazole, D_2_O exchangeable); ^13^C NMR (DMSO-*d_6_*) δc (ppm): 11.12, 14.82, 18.47, 107.65, 110.55, 115.74, 120.81, 129.12, 130.11, 131.17, 132.45, 134.59, 135.25, 136.25, 141.64, 142.87, 143.97, 151.49, 154.49, 157.66, 162.71, 166.38; EI-MS 516 [M^+^, 23%]; Anal. Calcd. for C_26_H_22_ClN_7_OS: C, 60.52; H, 4.30; Cl, 6.87; N, 19.00. Found: C, 60.31; H, 4.20; Cl, 6.87; N, 18.89.

*3-(4-chlorophenyl)-2-((1-(2-(furan-2-yl)-4-methyl-1H-imidazol-5-yl)ethylidene)hydrazineylidene)-4-methyl-5-(p-tolyldiazenyl)-2,3-dihydrothiazole* (**6e**). Color: Brownish red crystals (EtOH); yield (87%); m.p. 112–113 °C. IR (KBr, υ_max_, cm^−1^): 3322 (NH), 1594 (C=N); ^1^HNMR (300 MHz, DMSO-*d_6_*) δ (ppm): 2.17 (s, 3H, Ph–CH_3_), 2.36 (s, 3H, thiazole–CH_3_), 2.53 (s, N=C–CH_3_) 2.57 (s, imidazole–CH_3_), 6.62–6.65 (dd, *J* = 6, 3 Hz, 1H–furan), 6.86–6.87 (d, *J* = 3 Hz, 1H–furan), 7.27–7.44 (m, 6H–Ar), 7.58–7.64 (dd, *J* = 9, 6 Hz, 2H–Ar), 7.93–7.95 (d, *J* = 6 Hz, 1H–furan), 13.19 (s, 1H, NH–imidazole, D_2_O exchangeable); ^13^C NMR (DMSO-*d_6_*) δc (ppm): 11.72, 12.97, 15.68, 19.31, 108.20, 110.98, 115.91, 121.15, 128.37, 129.32, 130.85, 132.32, 134.61, 136.14, 137.58, 141.85, 143.06, 144.16, 150.94, 152.72, 157.85, 163.16, 166.64; EI-MS 530 [M^+^, 40%]; Anal. Calcd. for C_27_H_24_ClN_7_OS: C, 61.18; H, 4.56; N, 18.50. Found: C, 60.92; H, 4.42; N, 18.32.

*3-(4-chlorophenyl)-5-((4-chlorophenyl)diazenyl)-2-((1-(2-(furan-2-yl)-4-methyl-1H-imidazol-5-yl)ethylidene)hydrazineylidene)-4-methyl-2,3-dihydrothiazole* (**6f**). Color: Brownish red crystals (EtOH); yield (81%); m.p. 120–121 °C. IR (KBr, υ_max_, cm^−1^): 3355 (NH), 1565 (C=N); ^1^HNMR (300 MHz, DMSO-*d_6_*) δ (ppm): 2.17 (s, 3H, thiazole–CH_3_), 2.53 (s, N=C–CH_3_) 2.57 (s, imidazole-CH_3_), 6.86–6.89 (t, 1H–furan), 7.00–7.03 (d, *J* = 9 Hz, 1H–furan), 7.27–7.44 (m, 6H–Ar), 7.58–7.67 (dd, *J* = 12, 6 Hz, 2H–Ar), 7.92–7.95 (d, *J* = 9 Hz, 1H–furan), 14.12 (s, 1H, NH-imidazole, D_2_O exchangeable); ^13^C NMR (DMSO-*d_6_*) δc (ppm): 11.30, 12.87, 15.84, 107.36, 111.65, 115.38, 125.22, 126.44, 128.85, 130.07, 132.34, 135.27, 136.40, 138.26, 141.16, 142.42, 143.54, 151.29, 153.19, 157.33, 162.44, 166.29; EI-MS 550 [M^+^, 35%]; Anal. Calcd. for C_26_H_21_Cl_2_N_7_OS: C, 56.73; H, 3.85; N, 17.81. Found: C, C, 56.61; H, 3.73; N, 17.72.

#### 3.2.3. Synthesis of Dihydrothiazole Derivatives **8a** and **8b**

A solution containing carbothioamide derivatives **3a** or **3b** (1 mmol, 0.353 or 0.374g), chloroacetone (1 mmol, 0.0925 g), and sodium acetate (3 mmol, 0.246 g) in absolute ethanol (25 mL) was heated under reflux for 2–3 h (TLC). The precipitate formed was obtained by filtering, washing in ethanol and recrystallizing from EtOH to give compounds **8a** and **8b**.

*2-((1-(2-(furan-2-yl)-4-methyl-1H-imidazol-5-yl)ethylidene)hydrazineylidene)-4-methyl-3-(p-tolyl)-2,3-dihydrothiazole* (**8a**). Color: gray crystal (EtOH); yield (88%); m.p. 130–132 °C. IR (KBr, υ_max_, cm^−1^): 3343 (NH), 1523 (C=N); ^1^HNMR (300 MHz, DMSO-*d_6_*) δ (ppm): 1.84 (s, 3H, thiazole-CH_3_), 2.13 (s, 3H, Ph-CH_3_), 2.29 (s, N=C–CH_3_), 2.38 (s, imidazole–CH_3_), 6.12 (s, 1H-thiazole), 6.75–6.79 (dd, *J* = 9, 3 Hz, 1H–furan), 7.14–7.16 (d, *J* = 6 Hz, 1H–furan), 7.30–7.55 (m, 4H–Ar), 7.87–7.88 (d, *J* = 3 Hz, 1H–furan), 13.17 (s, 1H, NH-imidazole, D_2_O exchangeable); ^13^C NMR (DMSO-*d_6_*) δc (ppm): 12.56, 14.70, 14.95, 20.74, 96.85, 106.02, 111.77, 116.83, 124.50, 128.24, 128.69, 129.74, 134.72, 135.13, 137.55, 142.48, 143.83, 157.92, 170.51(C=N); EI-MS 391 [M^+^, 55%]; Anal. Calcd. for C_21_H_21_N_5_OS: C, 64.43; H, 5.41; N, 17.89. Found: C, 64.27; H, 5.34; N, 17.78.

*3-(4-chlorophenyl)-2-((1-(2-(furan-2-yl)-4-methyl-1H-imidazol-5-yl)ethylidene)hydrazineylidene)-4-methyl-2,3-dihydrothiazole* (**8b**). Color: gray crystal (EtOH); yield (85%); m.p. 140–141 °C. IR (KBr, υ_max_, cm^−1^): 3347 (NH), 1596 (C=N); ^1^HNMR (300 MHz, DMSO-*d_6_*) δ (ppm): 2.13 (s, 3H, thiazole–CH_3_), 2.38 (s, N=C–CH_3_), 2.58 (s, imidazole–CH_3_), 6.27 (s, 1H–thiazole), 6.76–6.81 (dd, *J* = 9, 6 Hz, 1H–furan), 7.09–7.12 (d, *J* = 9 Hz, 1H–furan), 7.35–7.55 (m, 4H–Ar), 7.87–7.88 (d, *J* = 3 Hz, 1H–furan), 14.02 (s, 1H, NH–imidazole, D_2_O exchangeable); ^13^C NMR (DMSO-*d_6_*) δc (ppm): 12.49, 13.85, 20.87, 94.83, 107.45, 110.30, 114.05, 130.07, 131.08, 135.01, 136.40, 137.05, 138.21, 141.36, 142.38, 151.22, 156.21, 166.27 (C=N); EI-MS 411 [M^+^,17%]; Anal. Calcd. for C_20_H_18_ClN_5_OS: C, 58.32; H, 4.40; N, 17.00. Found: C, 58.16; H, 4.34; N, 16.87.

#### 3.2.4. Synthesis of Thiazolidin-4-One Derivatives **10a** and **10b**

A mixture of carbothioamide derivatives **3a** or **3b** (1 mmol, 0.353 or 0.374 g), ethyl bromoacetate (1 mmol, 0.167 g), and sodium acetate (3 mmol, 0.246 g) in absolute ethanol (25 mL) was subjected to reflux for 2–3 h (TLC). The precipitate was acquired using filtration, followed by ethanol washing and recrystallization from EtOH, resulting in the formation of compounds **10a** and **10b**.

*2-((1-(2-(furan-2-yl)-4-methyl-1H-imidazol-5-yl)ethylidene)hydrazineylidene)-3-(p-tolyl)thiazolidin-4-one* (**10a**). Color: dark gray crystal (EtOH); yield (82%); m.p. 190–191 °C. IR (KBr, υ_max_, cm^−1^): 3347 (NH), 1741 (CO), 1596 (C=N); ^1^HNMR (300 MHz, DMSO-*d_6_*) δ (ppm): 2.17 (s, 3H, Ph–CH_3_), 2.37 (s, N=C–CH_3_), 2.53 (s, imidazole–CH_3_), 4.05 (s, 2H, thiazole–CH_2_), 6.58–6.61 (dd, *J* = 6, 3 Hz, 1H–furan), 6.82–6.83 (d, *J* = 3 Hz, 1H–furan), 7.09–7.14 (dd, *J* = 9, 6 Hz, 2H–Ar), 7.26–7.30 (dd, *J* = 9, 6 Hz, 2H–Ar), 7.73–7.74 (d, *J* = 3 Hz, 1H–furan), 13.26 (s, 1H, NH–imidazole, D_2_O exchangeable); ^13^C NMR (DMSO-*d_6_*) δc (ppm): 12.01, 20.10, 20.87, 34.69, 107.85, 110.05, 115.77, 128.27, 129.73, 136.10, 137.38, 139.75, 141.41, 142.63, 151.78, 157.98, 166.96, 172.95; EI-MS 393 [M^+^, 65%]; Anal. Calcd. for C_20_H_19_N_5_O_2_S: C, 61.05; H, 4.87; N, 17.80. Found: C, 60.92; H, 4.72; N, 17.68.

*3-(4-chlorophenyl)-2-((1-(2-(furan-2-yl)-4-methyl-1H-imidazol-5-yl)ethylidene)hydrazineylidene)thiazolidin-4-one* (**10b**). Color: light gray crystals (EtOH); yield (80%); m.p. 205–207 °C. IR (KBr, υ_max_, cm^−1^): 3345 (NH), 1743 (CO), 1590 (C=N); ^1^HNMR (300 MHz, DMSO-*d_6_*) δ (ppm): 2.37 (s, N=C–CH_3_), 2.54 (s, imidazole–CH_3_), 4.05 (s, 2H, thiazole–CH_2_), 6.81–6.85 (dd, *J* = 9, 6 Hz, 1H–furan), 7.10–7.12 (d, *J* = 6 Hz, 1H–furan), 7.30–7.33 (dd, *J* = 6, 3 Hz, 2H–Ar), 7.55–7.59 (dd, *J* = 9, 6 Hz, 2H–Ar), 7.70–7.71 (d, *J* = 3 Hz, 1H–furan), 14.48 (s, 1H, NH–imidazole, D_2_O exchangeable); ^13^C NMR (DMSO-*d_6_*) δc (ppm): 12.34, 20.31, 35.10, 108, 110.51, 115.59, 128.87, 131.04, 135.16, 136.40, 137.67, 141.74, 142.79, 152.03, 158.43, 167.64, 173.70; EI-MS 413 [M^+^, 90%]; Anal. Calcd. for C_19_H_16_ClN_5_O_2_S: C, 55.14; H, 3.90; N, 16.92. Found: C, 55.02; H, 3.87; N, 16.78.

### 3.3. Antioxidant Activity 

The total antioxidant capacity (TAC) of each compound was evaluated according to the method described by Prieto et al. [54]. An aliquot of 100 μL of sample solution was combined with 900 μL of reagent solution (0.6 M sulfuric acid, 28 mM sodium phosphate, and 4 mM ammonium molybdate) (Sigma-Aldrich, Tokyo, Japan). For the blank, 100 μL of deionized water was used in place of the sample. The tubes were incubated in a boiling water bath at 95 °C for 90 min. After the samples were cooled to room temperature, the absorbance of the aqueous solution in each sample was measured at 695 nm in the spectrophotometer (Shimadzu UV1024-PC, Burlington, MA, USA). A standard curve of ascorbic acid (0.2–1 mg/mL) has been constructed to determine the total antioxidant equivalent. 

### 3.4. Test Microbes Used

*Staphylococcus aureus* ATCC 6538 (G + ve bacteria) and *Escherichia coli* ATCC 25933 (G -ve bacteria) and *Candida albicans* ATCC 10231 (yeast) were used and grown on Mueller Hinton medium.

### 3.5. Antimicrobial Activities

The antimicrobial activity of produced compounds was studied by the cup diffusion agar method. The four representative test microbes used were *Staphylococcus aureus* ATCC 6538-P (G +ve), *Escherichia coli* ATCC 25933 (G − ve), *Candida albicans* ATCC 10231 (yeast), and *Aspergillus niger* NRRL-A326 (fungus). Nutrient agar plates were heavily inoculated regularly with 0.1 mL of 10^5^–10^6^ cells/mL in the case of bacteria and yeast. Potato dextrose agar plates seeded with 0.1 mL (10^6^ cells/mL) of the fungal inoculum were used to evaluate the antifungal activities. In total, 100 µL of samples dissolved in DMSO (10 mg in 2 mL DMSO) were placed in initiated holes in inoculated plates. Then, plates were kept at a low temperature (4 °C) for 2–4 h to allow maximum diffusion. The plates were then incubated at 37 °C for 24 h for bacteria and at 30 °C for 48 h in an upright position to allow maximum growth of the organisms. The antimicrobial activity of the test agent was determined by measuring the diameter of the zone of inhibition expressed in millimeters (mm). The experiment was carried out more than once, and the mean reading was recorded. Neomycin and cyclohexamide were used as antibacterial and antifungal standards, respectively.

### 3.6. Preparation of Bacterial Culture

Bacterial cultures were prepared under sterile conditions by insulating a 100 mL bottle with each test microbe, capped, and incubated at 35 °C for 24 h. Clean bacterial cells were prepared by centrifuging the growth culture, under sterile conditions, in a cooling centrifuge at 400 rpm for 15 min. The bacterial cells were resuspended in 20 mL of sterile normal saline and centrifuged again at 4000 rpm for 5 min. This step was repeated until the supernatant was clear. The pellet was then suspended in 20 mL of sterile normal saline. The optical density of the bacterial suspension was recorded at 500 nm, and serial dilutions were carried out with appropriate aseptic techniques until the optical density was in the range of 0.5–1.0. The actual number of colony-forming units was carried out to obtain a concentration of 5 × 10^6^ cfu/mL.

### 3.7. Preparation of Resazurin Solution

The resazurin solution was prepared by dissolving 675 mg in 100 mL of sterile distilled water and shaking well with a vortex mixer and was sterilized by filtration through a membrane filter (pore size of 0.22–0.45 µm).

### 3.8. Preparation of the Plates

Microplates, 96-well, were prepared and labeled under aseptic conditions. A volume of 500 µL of test material in DMSO (a stock concentration of 5 mg/mL for purified compounds) was pipetted into the first row of the plate. To all other wells, 50 µL of broth medium was added. Serial dilutions were performed. To each well, 10 µL of resazurin indicator solution was added, and 10 µL of bacterial suspension (5 × 10^6^ cfu/mL) was added to each well. Each plate was wrapped loosely with parafilm to ensure that bacteria did not become dehydrated. The plates were prepared in duplicate and placed in an incubator set at 37 °C for 18–24 h. The color change was then assessed visually. Any color changes from purple to pink or colorless were recorded as positive. The lowest concentration at which color change occurred was taken as the MIC value. Neomycin was used as a standard.

### 3.9. Molecular Docking Method

Using PyRx tools Autodock Vina (version 1.1.2) [55], the molecular docking investigation of the compounds under study with the crystal structure of *S. aureus* DNA gyrase B (PDB: 3U2D) and the X-ray crystallographic structure of *E. coli* DNA gyrase B (PDB: 1S14) was achieved. The original ligands and molecules of water were deleted from the proteins by means of the VEGA ZZ 2.3.2 tool, tracked by the addition of Kollman charges and polar hydrogen, and then transformed to PDBQT format using Autodock Vina tools. All planned compounds are saved as a mol file, protonated, minimized, and then transformed to a pdb file by means of Open Babel software version 2.3. The created pdb file was set up with Autodock Vina Tools to establish a number of torsions, and the same software was also used for pdbqt file creation. The grid map has been built by means of AutoGrid, together with a grid box. The number of docked poses generated for each compound was categorized according to the binding energy. The poses with the lowest binding energy and 0 Å RMSD (root mean square deviation) have been considered adequate and most complex with the receptor for investigation. Using BIOVIA Discovery Studio 2021, the molecular interactions and binding modes of the top poses were visually examined.

## 4. Conclusions

A set of 1,3-thiazole derivatives **6a**–**f**; **8a**,**b** and thiazolidinone derivatives **10a**,**b** were efficiently synthesized starting from carbothioamides **3a**,**b**. The developed compounds were identified using elemental and spectroscopic data. The compounds showed adequate antioxidant and antibacterial properties. Thiazole derivatives **6a** showed the best antioxidant activities at 2007.67 µgAAE/g dry sample. The majority of compounds exhibited strong antibacterial activity, with low minimum inhibitory concentration (MIC) values ranging between 4.88 and 78.125 µg/mL against *S. aureus* and *E. coli* and between 156.25 and 312.5 µg/mL against *C. albicans*. Identifying novel antimicrobial chemicals is a challenging endeavor, but through molecular docking studies, I am progressing toward discovering potent medicines. The study assessed how antimicrobial chemicals bind to the crystal structures of DNA gyrase B. A strong correlation was found between the in vitro and molecular docking experiments. All the compounds examined showed strong binding energy to the target DNA gyrase B enzymes, mimicking the interaction with the crucial amino acids of the target DNA gyrase B enzymes, similar to the natural inhibitor. The results are intriguing and provide exciting leads for the creation of novel and efficient antibacterial agents.

## Figures and Tables

**Table 1:** The total antioxidant activities were determined using the phosphomolybdate method.

Cpds No.	Total Antioxidant (µg AAE/g Dry Sample)		Cpds No.	Total Antioxidant (µg AAE/g Dry Sample)
**3a**	1962.48 ± 35.83		**6e**	1729.94 ± 4.44
**3b**	1369.14 ± 37.27		**6f**	1654.76 ± 3.39
**6a**	2007.67 ± 33.13		**8a**	1321.74 ± 11.13
**6b**	1731.98 ± 16.76		**8b**	1883.96 ± 15.57
**6c**	1676.06 ± 0.56		**10a**	1396.06 ± 1.47
**6d**	1770.69 ± 4.24		**10b**	1370.87 ± 3.70

**Table 2:** Antimicrobial activities of the produced compounds.

No	Cpds No.	Anti-Microbial Activity (mm)			
		*S. aureus*	*E. coli*	*C. albicans*	*A. niger*
1	**3a**	28 ± 0.29	27 ± 0.24	26 ± 0.20	0
2	**3b**	23 ± 0.20	18 ± 0.15	17 ± 0.12	16 ± 0.06
3	**6a**	0	0	0	0
4	**6b**	0	0	0	0
5	**6c**	0	0	0	37 ± 0.29
6	**6d**	23 ± 0.16	21 ± 0.10	26 ± 0.17	15 ± 0.31
7	**6e**	21 ± 0.12	16 ± 0.06	19 ± 0.45	16 ± 0.22
8	**6f**	20 ± 0.29	18 ± 0.15	21 ± 0.12	25 ± 0.17
9	**8a**	25 ± 0.60	28 ± 0.40	21 ± 0.30	0
10	**8b**	24 ± 0.17	25 ± 0.15	23 ± 0.12	0
11	**10a**	22 ± 0.08	25 ± 0.17	26 ± 0.10	0
12	**10b**	21 ± 0.10	24 ± 0.22	24 ± 0.20	0
13	Neomycin	29 ± 0.21	26 ± 0.23	28 ± 0.06	0
14	Cyclohexamide	0	0	0	34 ± 0.16

Values are given as mean ± standard error.

**Table 3:** Minimum inhibition concentration (MIC) and minimum bactericidal concentration (MBC) of the synthesized compounds against different test microbes.

Cpds No.	*S. aureus*	*E. coli*	*C. albicans*
	MIC (µg/mL)	MIC (µg/mL)	MIC (µg/mL)
**3a**	4.88	4.88	156.25
**3b**	19.53	9.77	156.25
**8a**	9.77	4.88	312.50
**8b**	78.125	9.77	156.25
**6d**	78.125	39.06	156.25
**6e**	78.125	19.53	156.25
**6f**	39.06	19.53	156.25
**10a**	19.53	9.77	312.5
**10b**	19.53	4.88	156.25
Neomycin	39.06	78.125	312.50

**Table 4:** Binding energy (Kcal/mol) and interactions of tested compounds against *S. aureus* DNA gyrase B (PDB: 3U2D) and *E. coli* DNA gyrase B (PDB: 1S14).

Target Protein (PDB)	Cpds No.	Binding Energy (Kcal/mol)
*S. aureus* DNA gyrase B (PDB: 3U2D)	**3a**	−8.4
	**3b**	−7.9
	**8a**	−8.1
	**8b**	−8.1
	**10b**	−8.3
	**08B**	−8.0
*E. coli* DNA gyrase B (PDB: 1S14)	**3a**	−6.8
	**3b**	−6.9
	**8a**	−6.6
	**8b**	−6.5
	**10b**	−6.5
	**NOV**	−8.3

**Table 5:** Types of interactions between the synthesized compounds and key amino acid residues of *S. aureus* DNA gyrase B (PDB: 3U2D) and *E. coli* DNA gyrase B (PDB: 1S14).

Target (PDB ID)	Cpd No.	Moieties from the Compound	Key Amino Acid Residues	Type of Interaction
*S. aureus* DNA gyrase B (PDB: 3U2D)	**3a**	SO_2_ NH	THR173 ASN54, ASP81	Hydrogen bond
	**8a**	-S- of thiazine moiety Furan moiety	ARG144 ASN54	Hydrogen bond
		Imidazole moiety	ASP81	Pi-anion
	**8b**	-S- of thiazine moiety	ARG144	Hydrogen bond
		Imidazole moiety	ASP81	Pi-anion
*E. coli* DNA gyrase B (PDB: 1S14)	**3a**	NH-NH	ASN1042, GLU1046	Hydrogen bond
		Furan moiety	ARG1072	Pi-anion
	**3b**	CO NH Imidazole moiety	THR1163 GLU1046 PRO1075	Hydrogen bond
		Furan moiety	ARG1072	Pi-anion
	**8a**	-S- of thiazine moiety Imidazole moiety	THR1163 GLU1046	Hydrogen bond
		Furan moiety	ARG1072	Pi-anion
		Thiazine moiety	ASN1042	Pi-stacked
	**8b**	-S- of thiazine moiety Imidazole moiety	THR1163 GLU1046	Hydrogen bond
		Imidazole moiety	ARG1072	Pi-anion
		Thiazine moiety	ASN1042	Pi-stacked
	**10b**	CO NH of imidazole moiety Imidazole moiety	THR1163 GLU1046 PRO1075	Hydrogen bond
		Furan moiety	ARG1072	Pi-anion

## Data Availability

Data are contained within the article and Supplementary Materials.

## Supplementary Materials

The following supporting information can be downloaded at: https://www.mdpi.com/article/10.3390/molecules29071491/s1.
