# Supplementary material for: Novel Thiazole Derivatives Containing Imidazole and Furan Scaffold: Design, Synthesis, Molecular Docking, Antibacterial, and Antioxidant Evaluation
Source: Molecules. 2024 Mar 27;29(7):1491. doi: 10.3390/molecules29071491 (PMC11013646; doi:10.3390/molecules29071491)
Supplement: Supplementary file 1 [file molecules-29-01491-s001.zip › molecules-2928153-supplementary.pdf]

Supplementary Data

Novel Thiazole Derivatives Containing Imidazole and Furan Scaffold: Design, Synthesis, Molecular Docking, Antibacterial, and Antioxidant Evaluation

Fatimah Agili

Department of Physical Sciences, Chemistry Division, College of Science, Jazan University, P.O. Box 114, Jazan, 45142, Kingdom of Saudi Arabia
E-mail: dr.fatmah2001@gmail.com

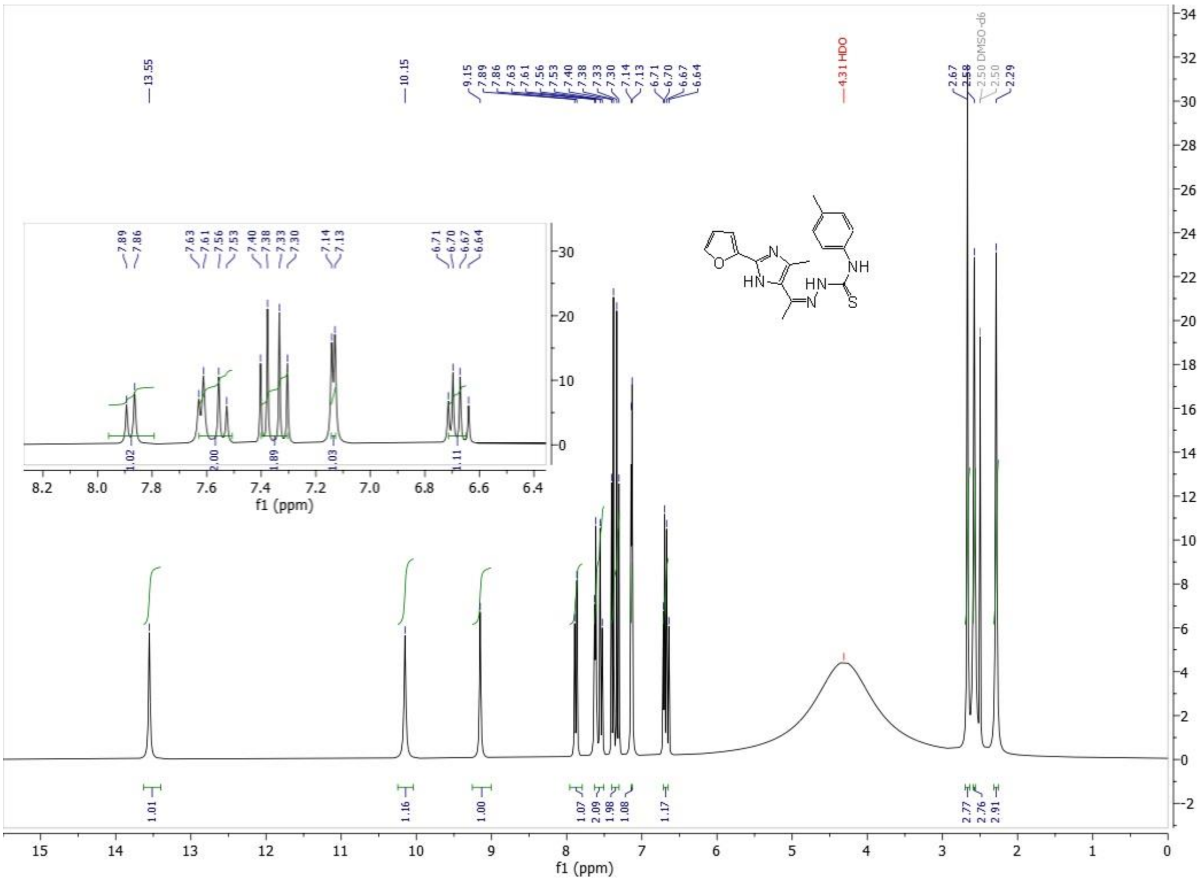

Figure S1. ^{1}H NMR Spectrum of compound **3a**

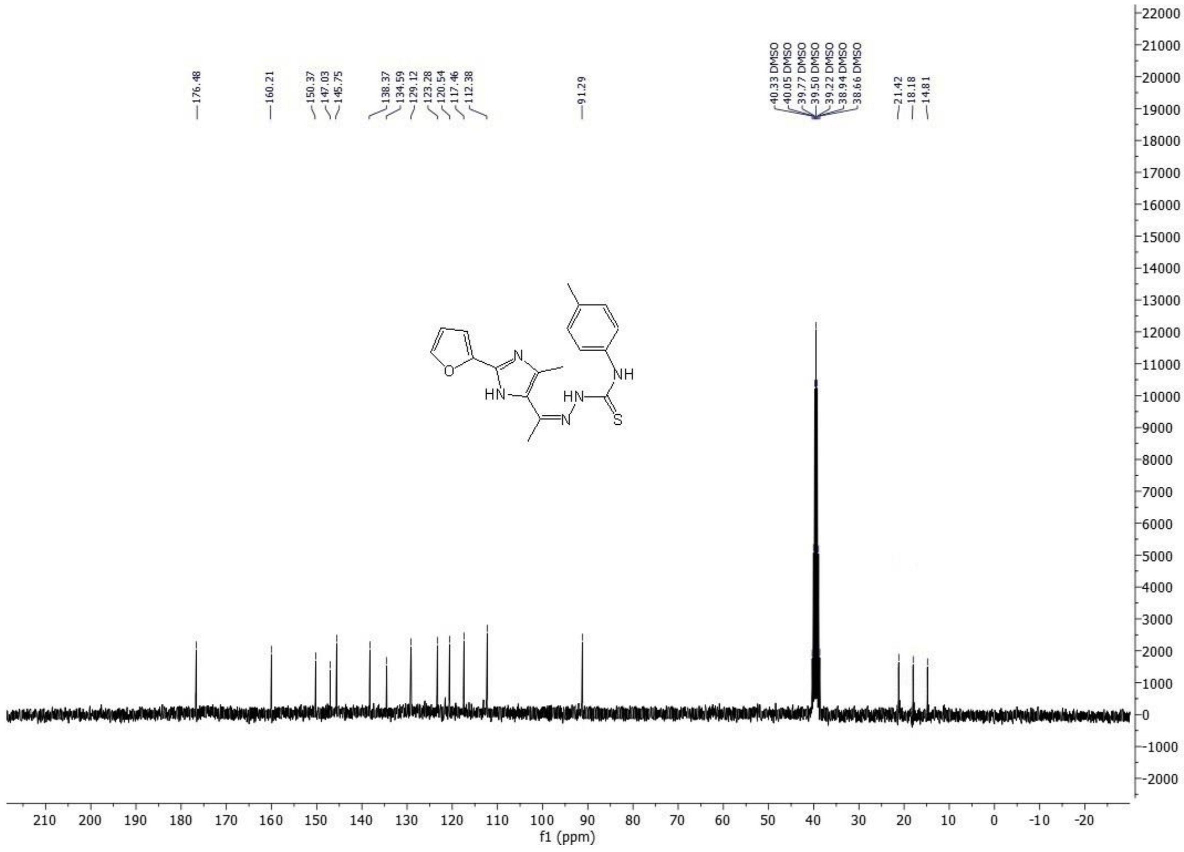

Figure S2. ^{13}C NMR Spectrum of compound **3a**

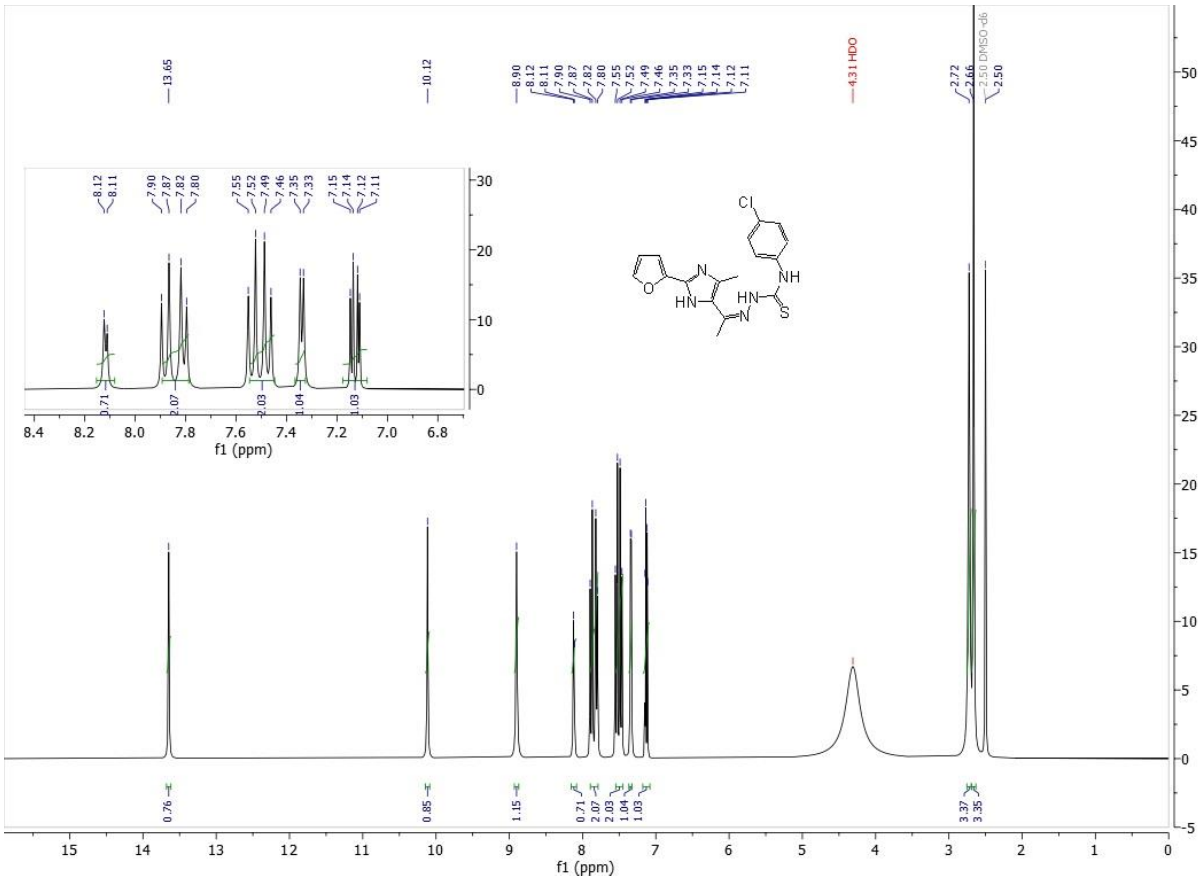

Figure S3. ^{1}H NMR Spectrum of compound **3b**

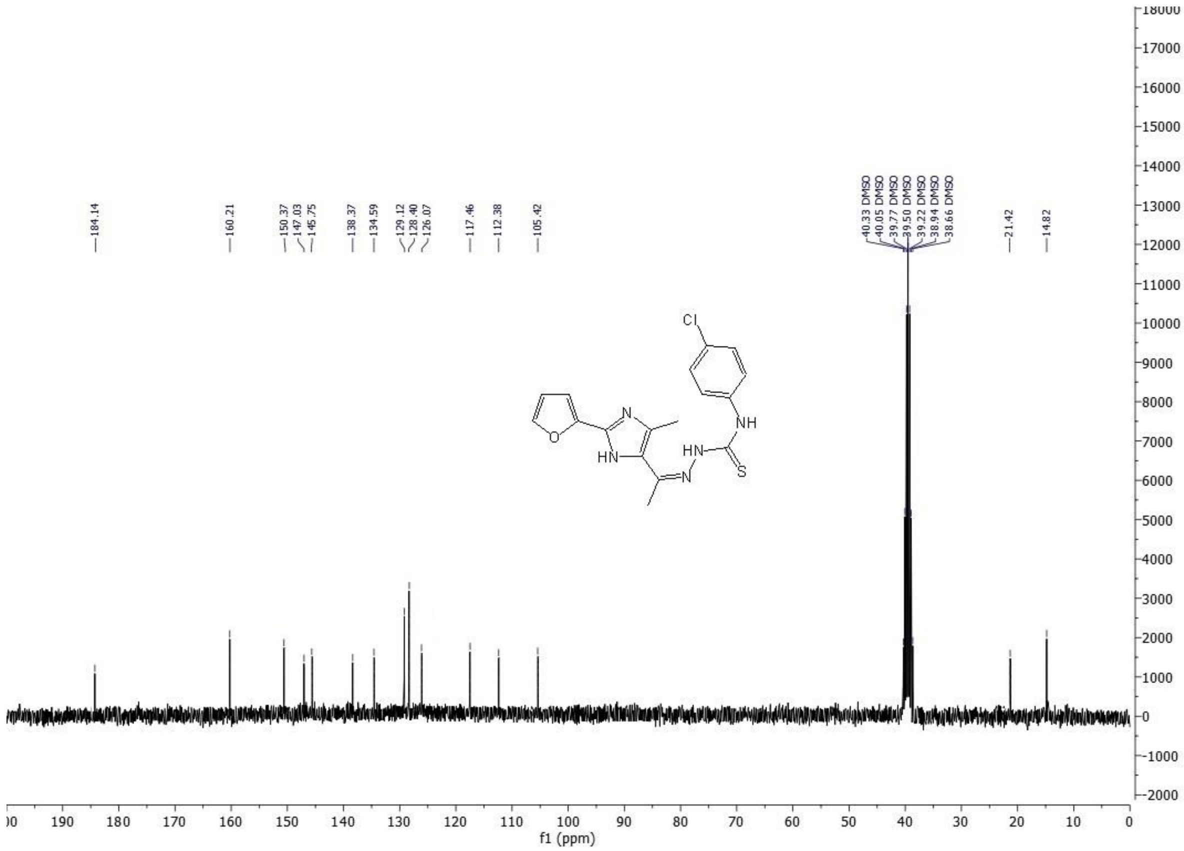

Figure S4. ^{13}C NMR Spectrum of compound **3b**

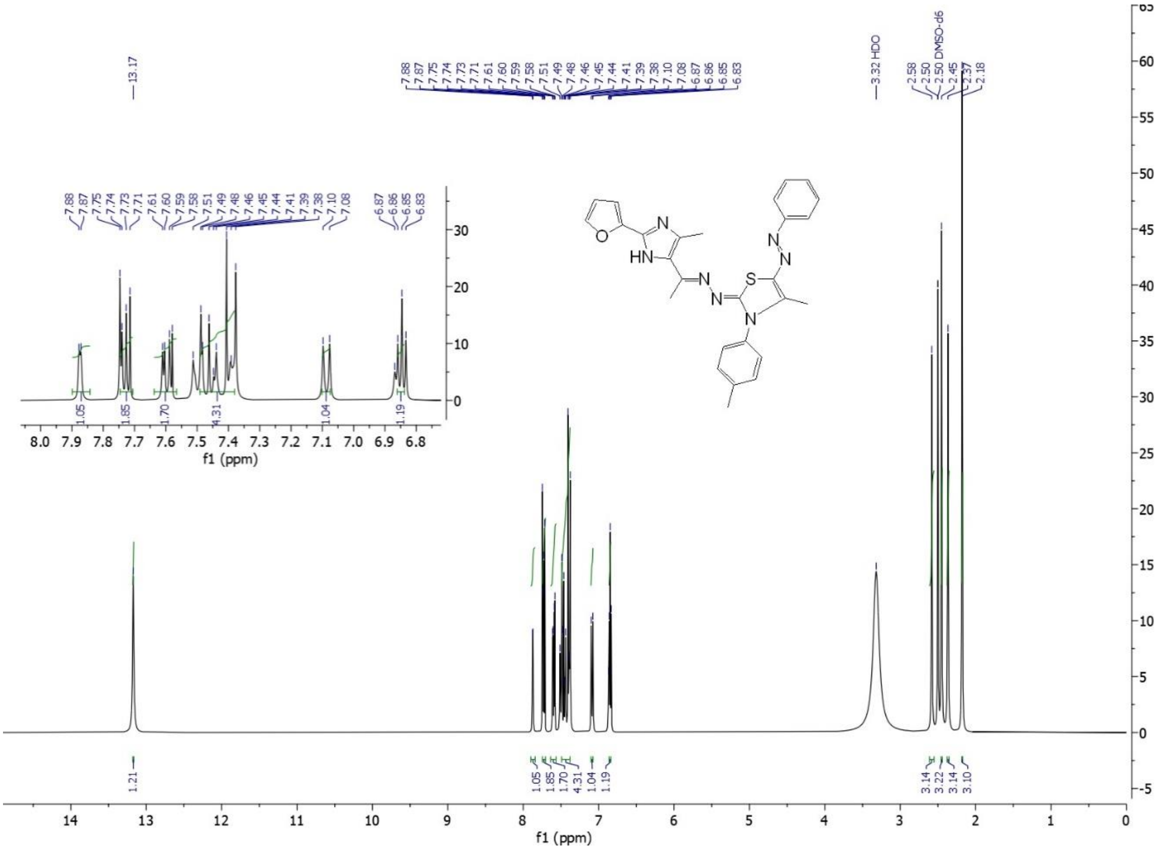

Figure S5. ^{1}H NMR Spectrum of compound **6a**

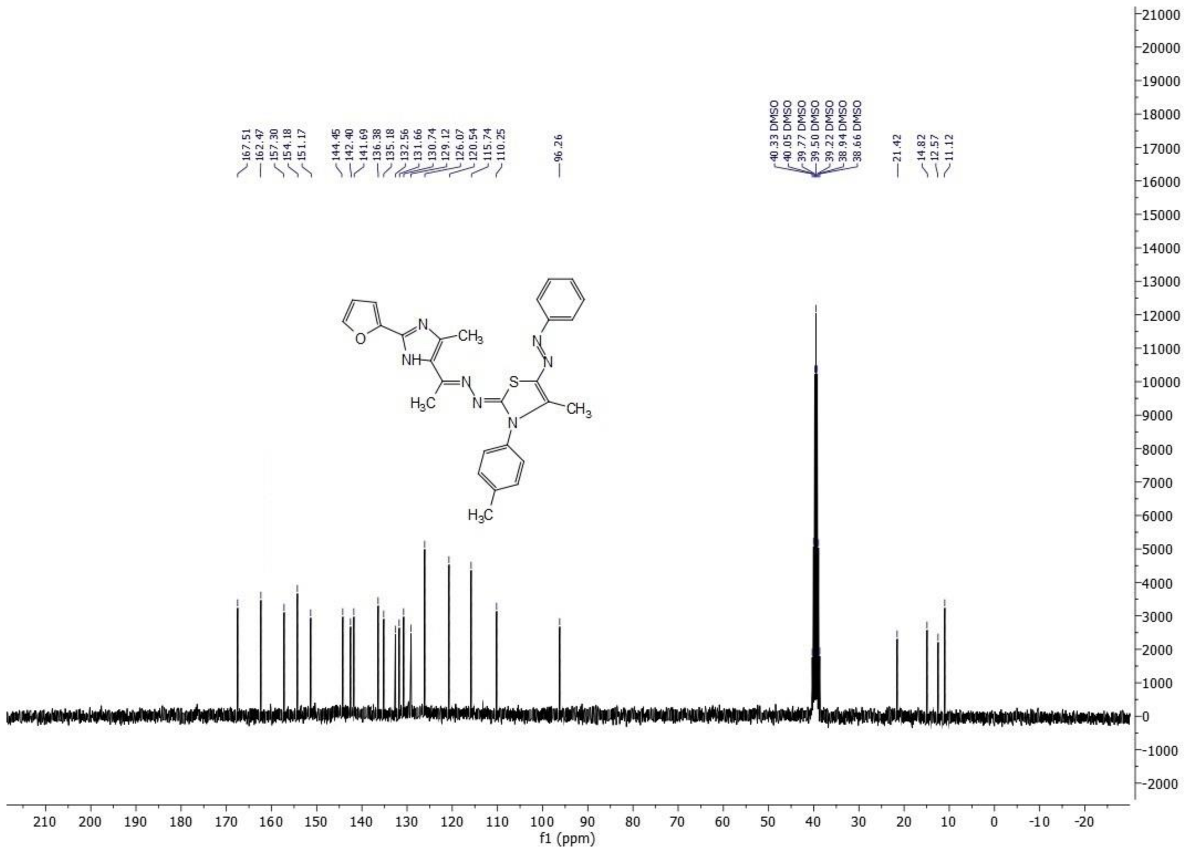

Figure S6. ^{13}C NMR Spectrum of compound **6a**

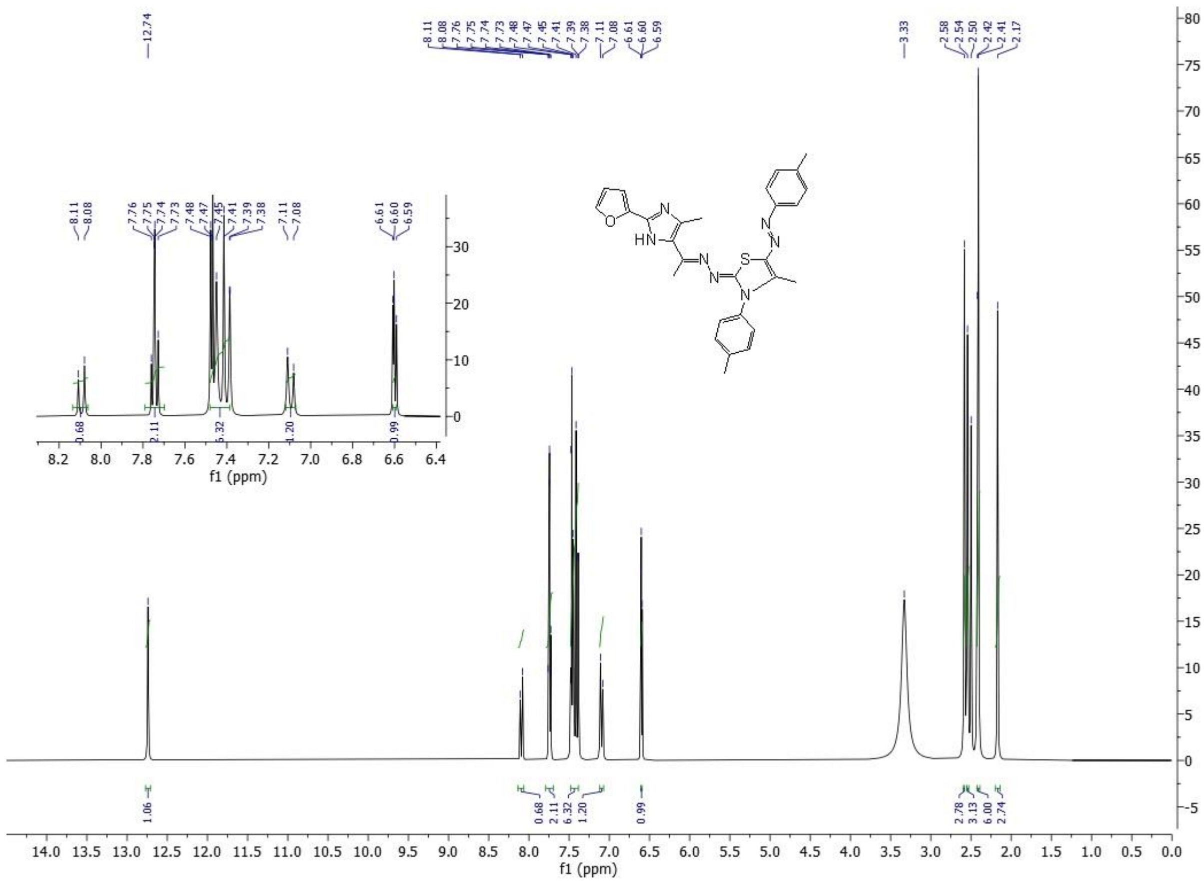

Figure S7. ^{1}H NMR Spectrum of compound **6b**

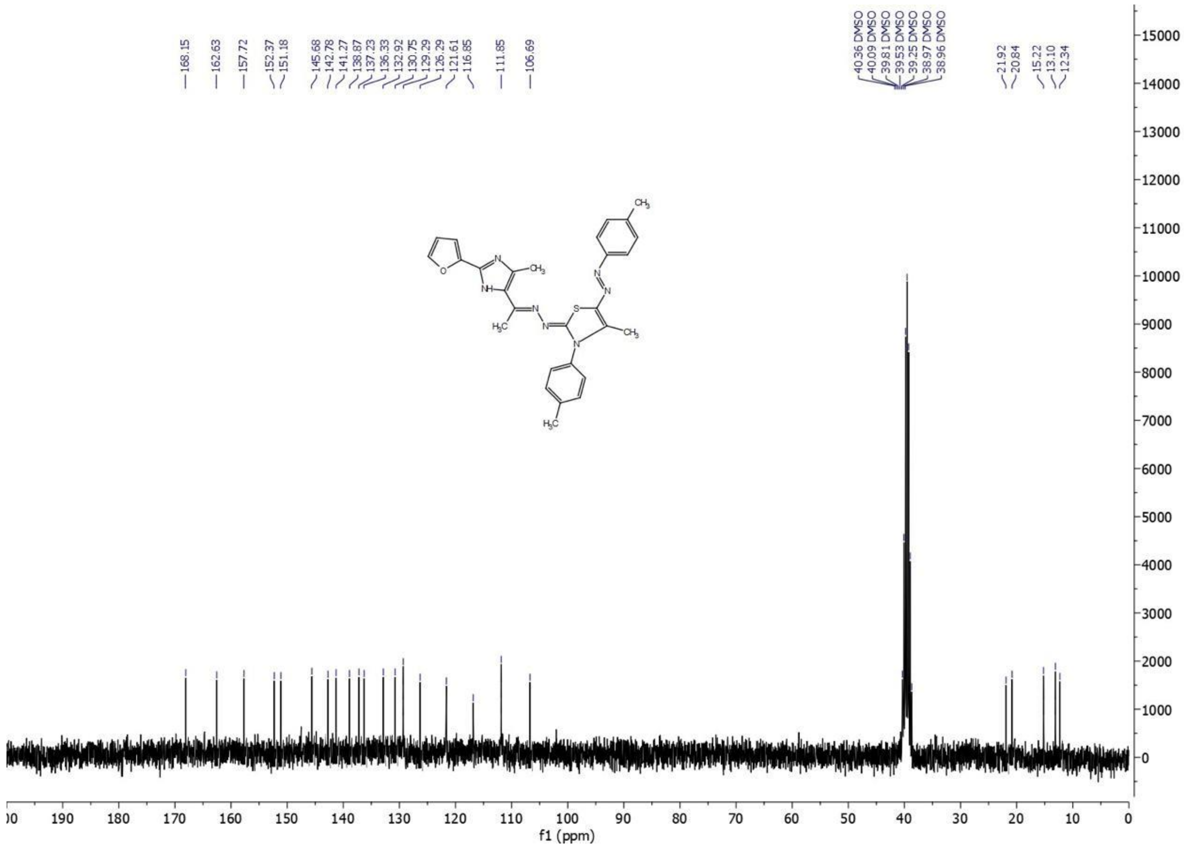

Figure S8. ^{13}C NMR Spectrum of compound **6b**

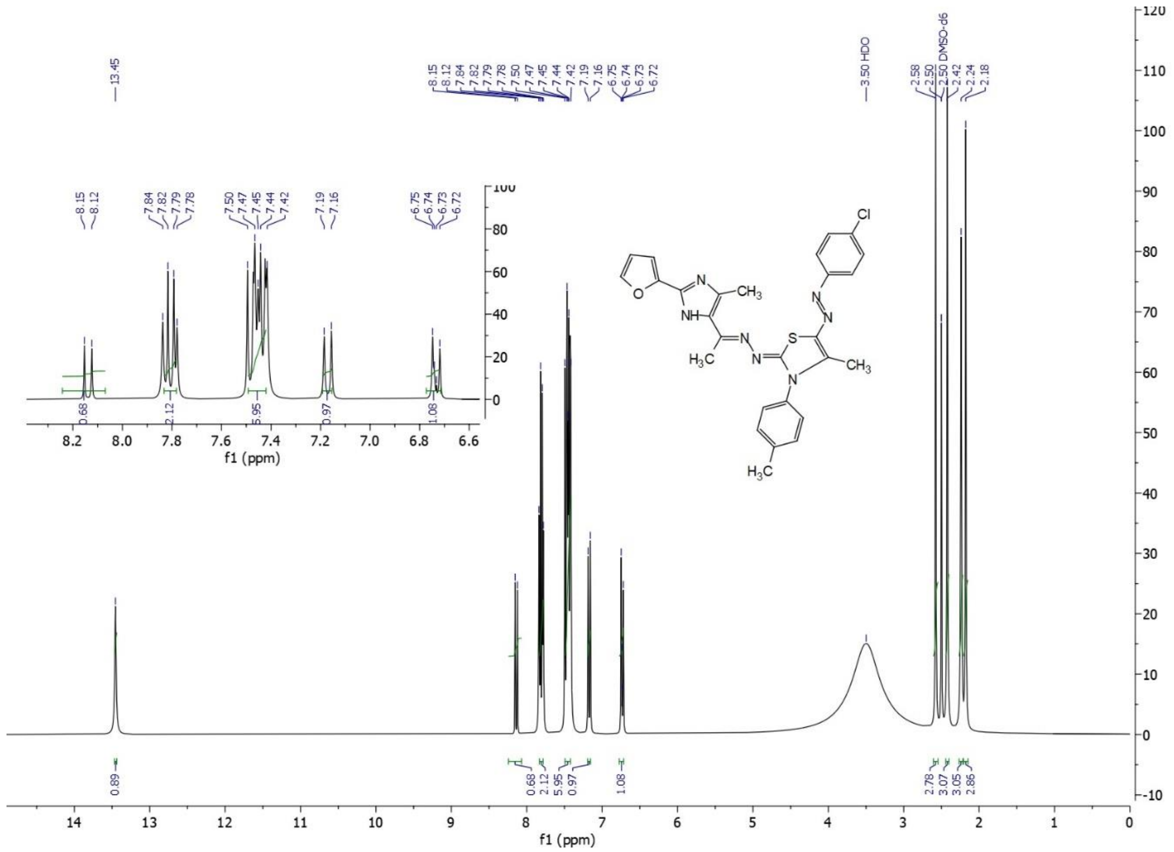

Figure S9. ^{1}H NMR Spectrum of compound **6c**

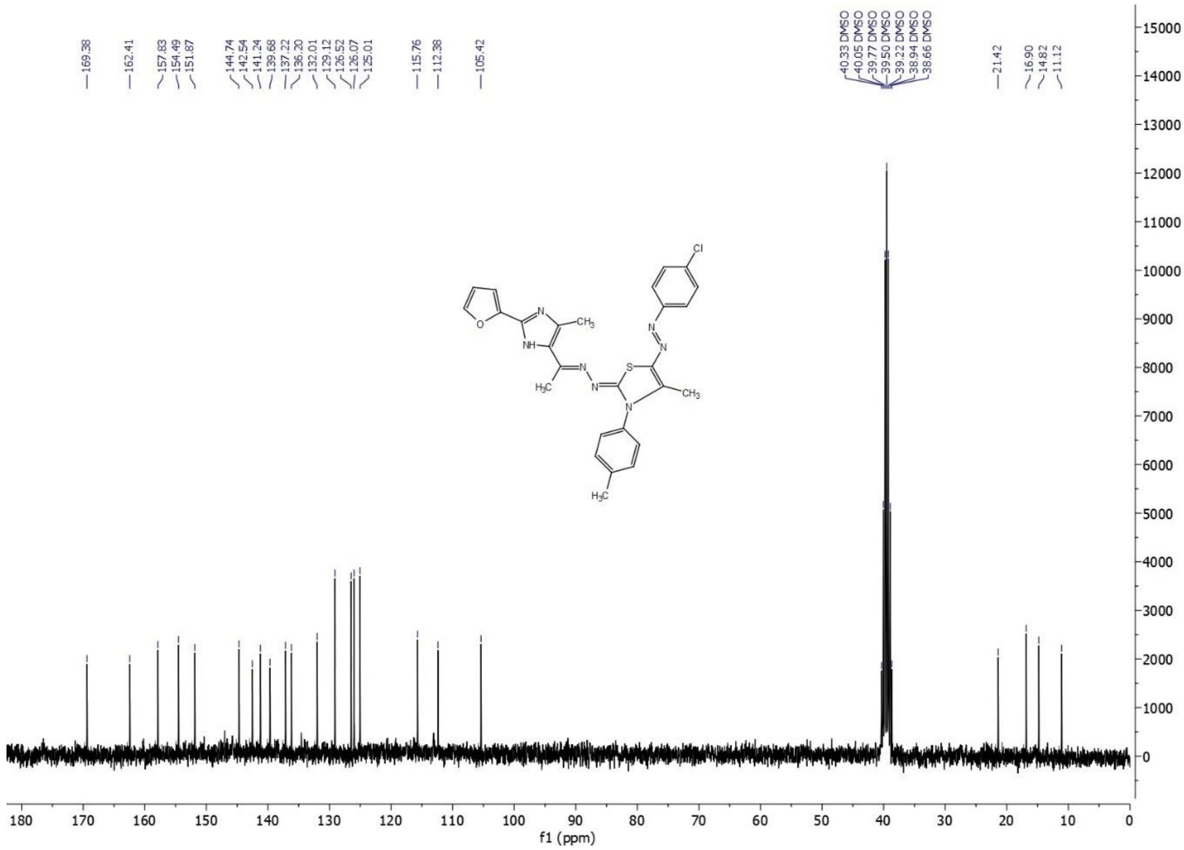

Figure S10. ^{13}C NMR Spectrum of compound **6c**

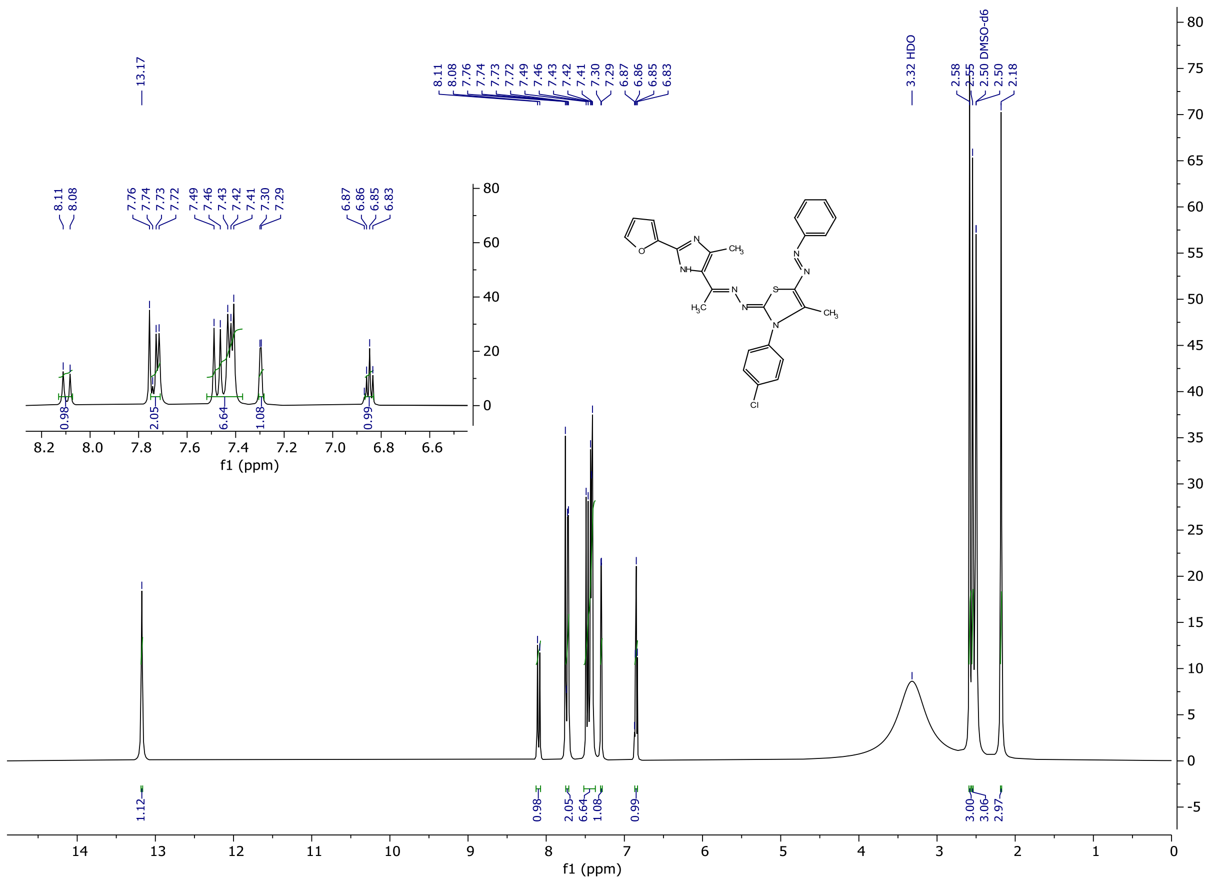

Figure S11. ^{1}H NMR Spectrum of compound **6d**

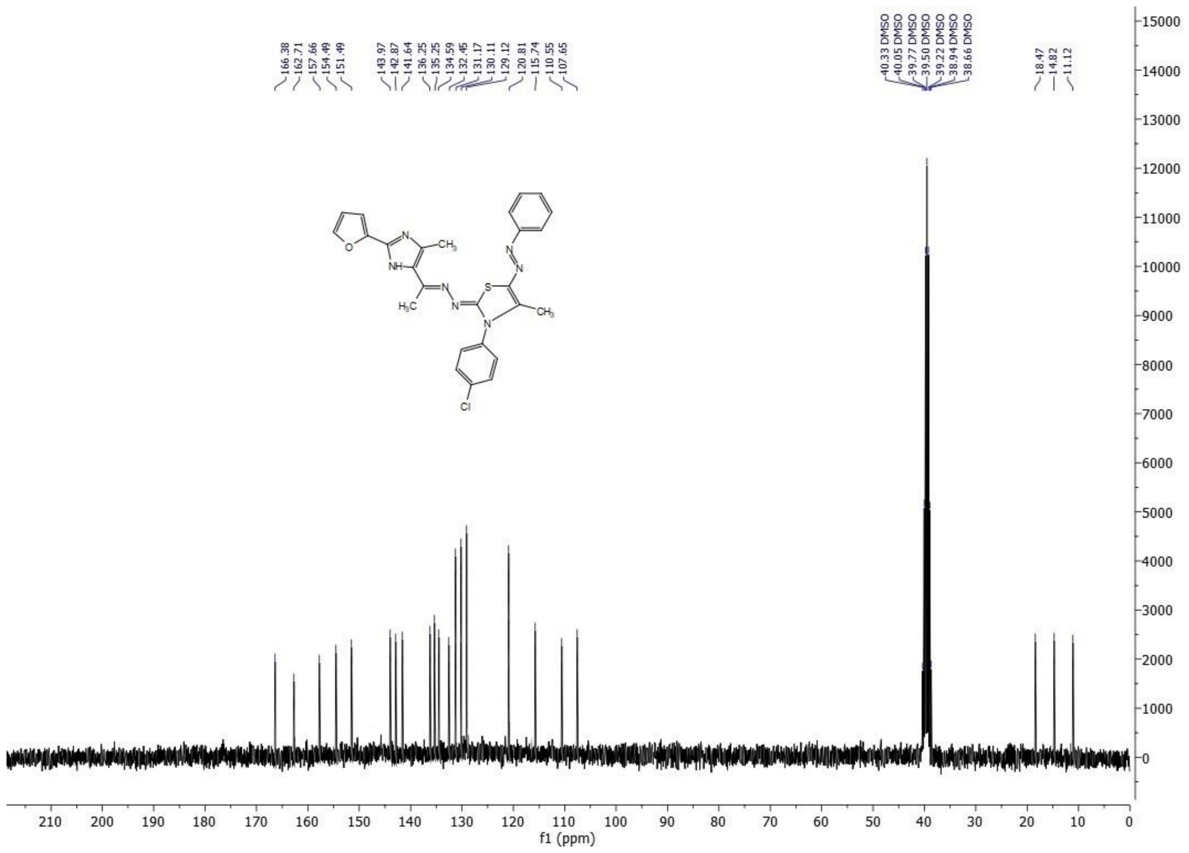

Figure S12. ^{13}C NMR Spectrum of compound **6d**

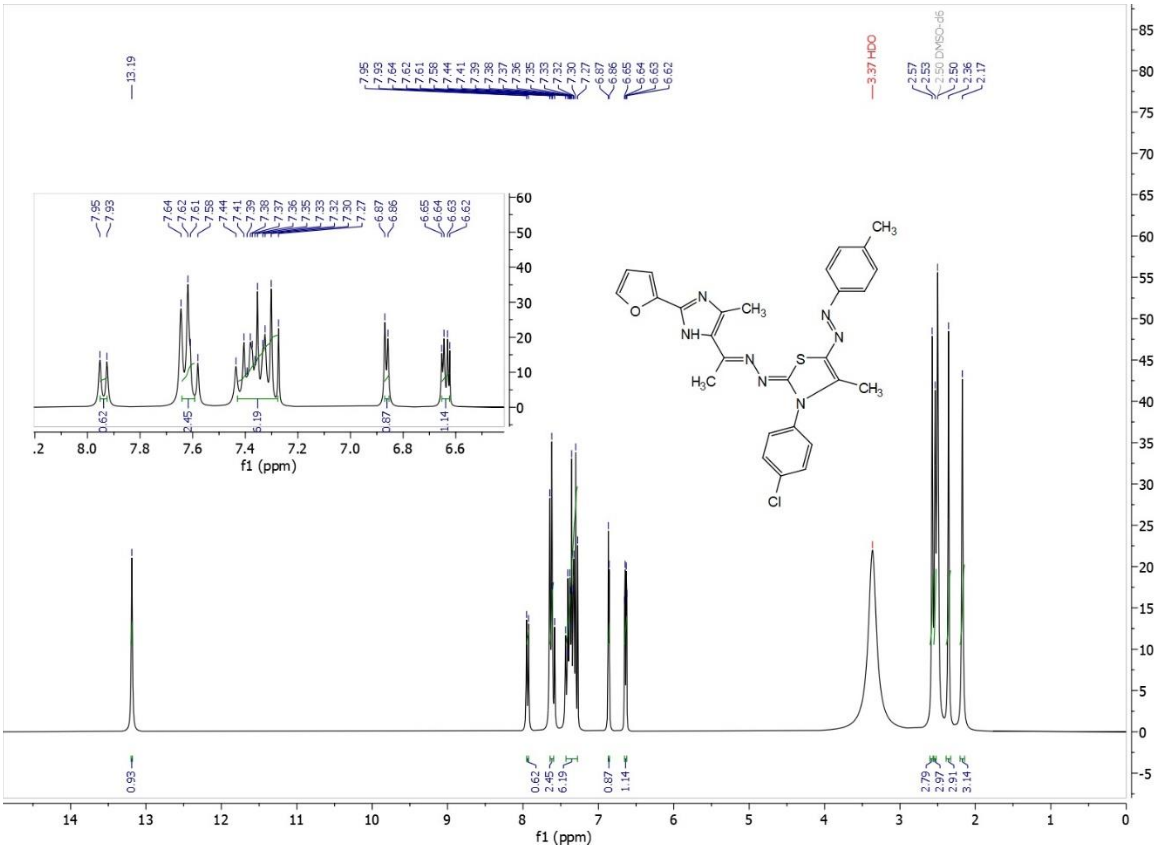

Figure S13. ^{1}H NMR Spectrum of compound **6e**

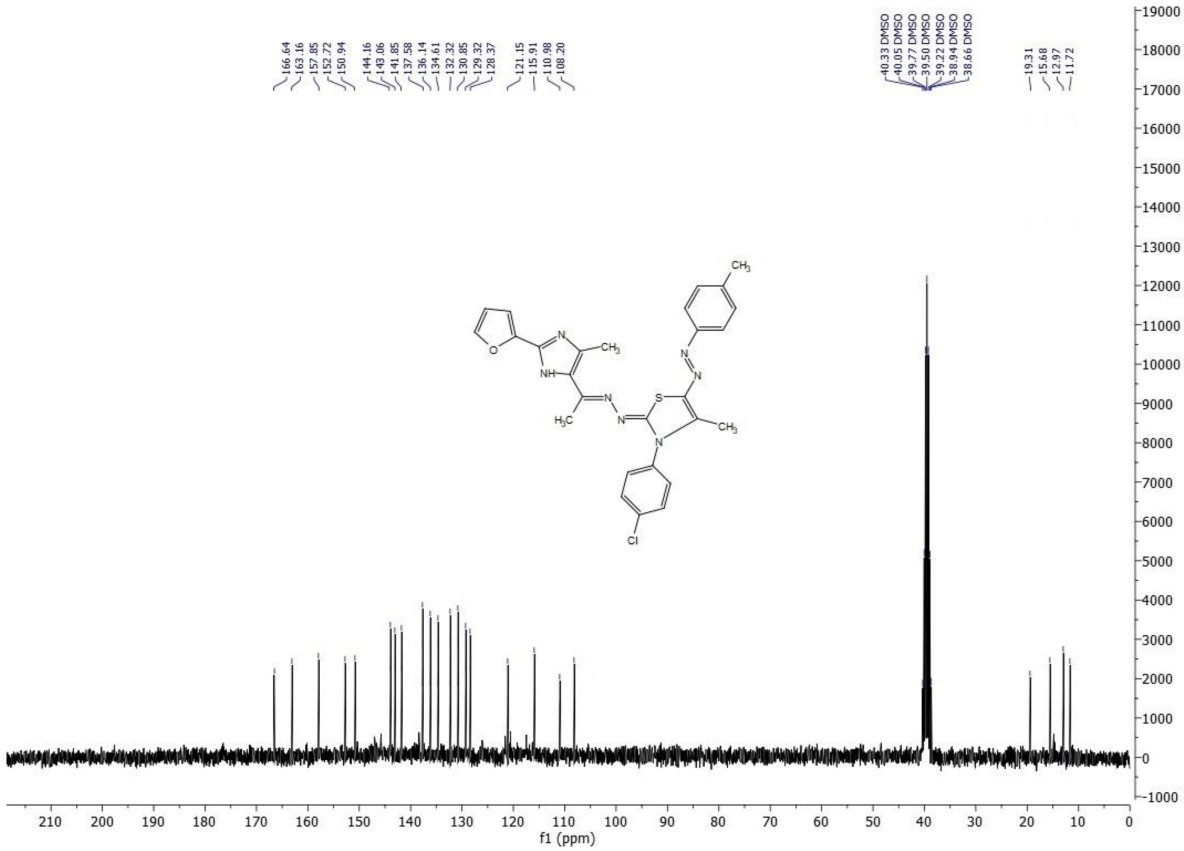

Figure S14. ^{13}C NMR Spectrum of compound **6e**

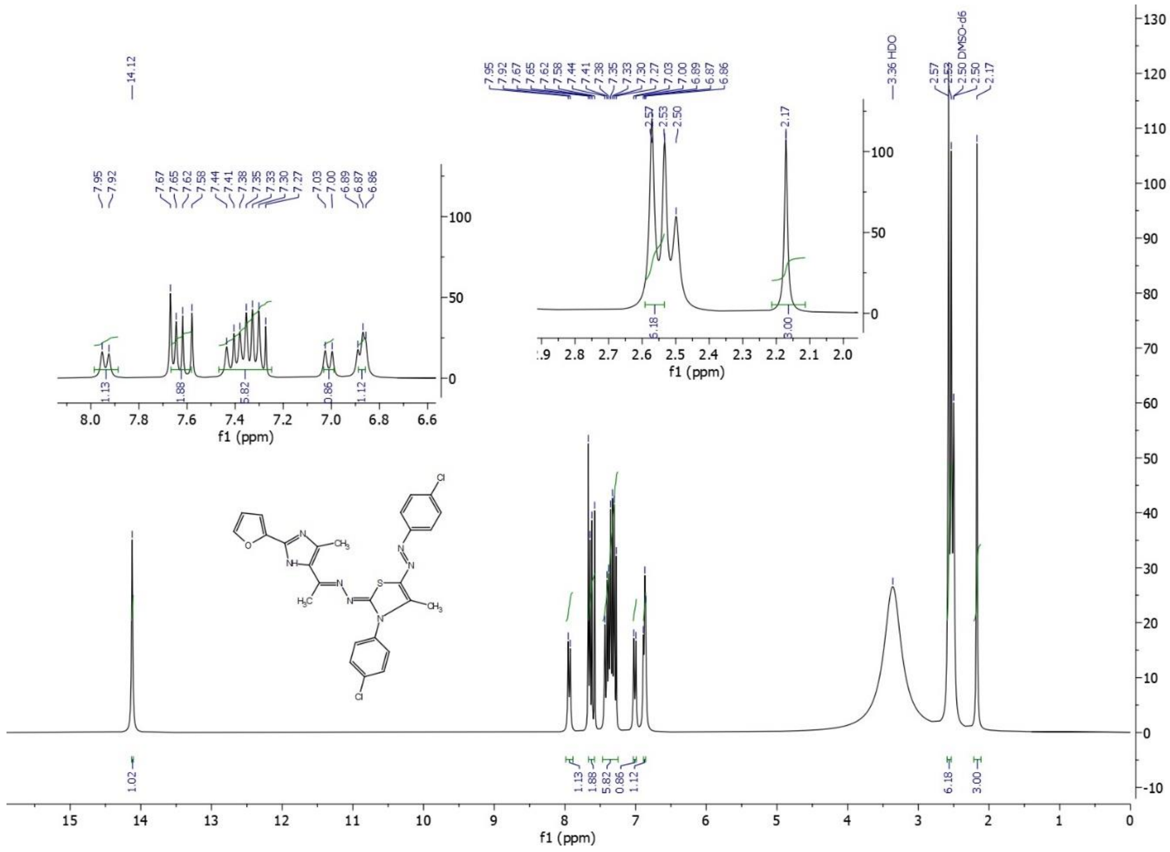

Figure S15. ^{1}H NMR Spectrum of compound **6f**

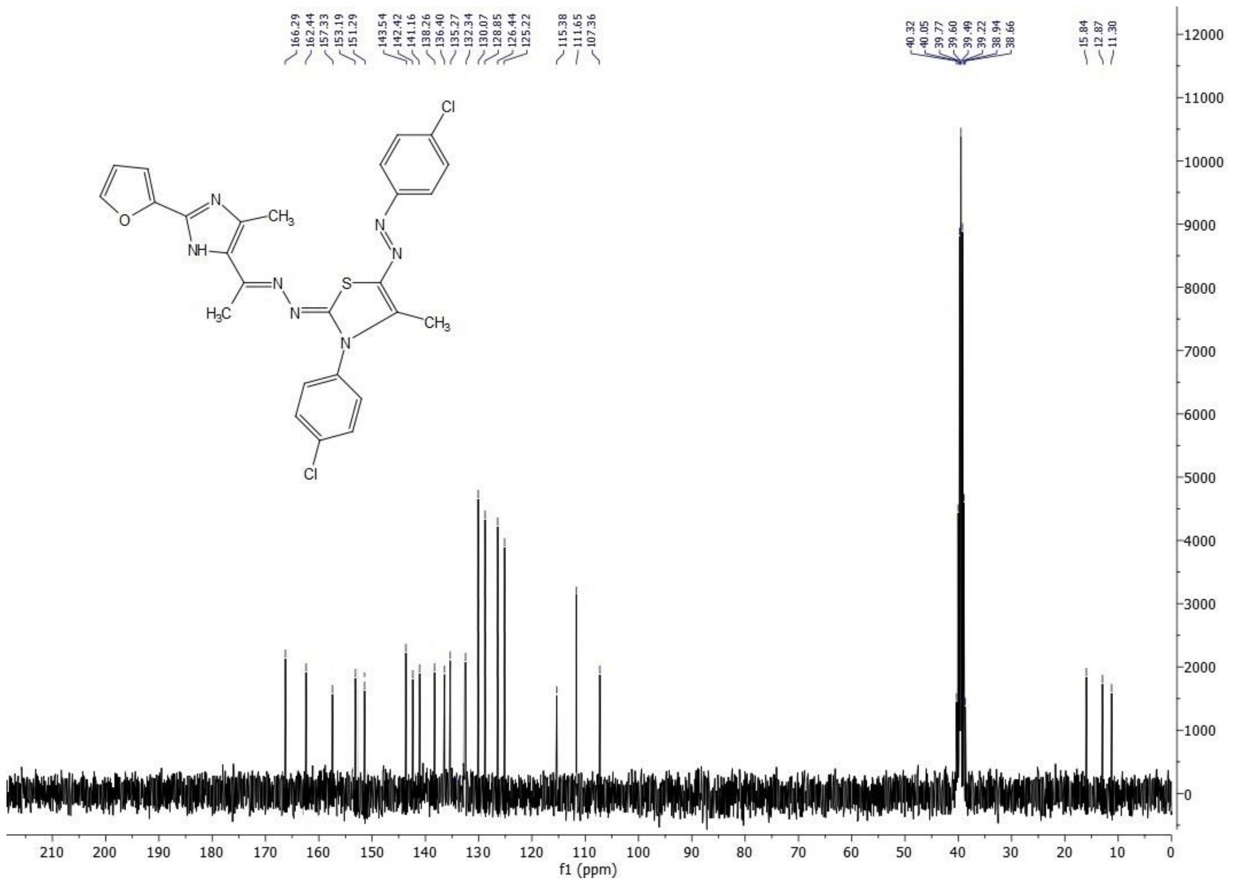

Figure S16. ^{13}C NMR Spectrum of compound **6f**

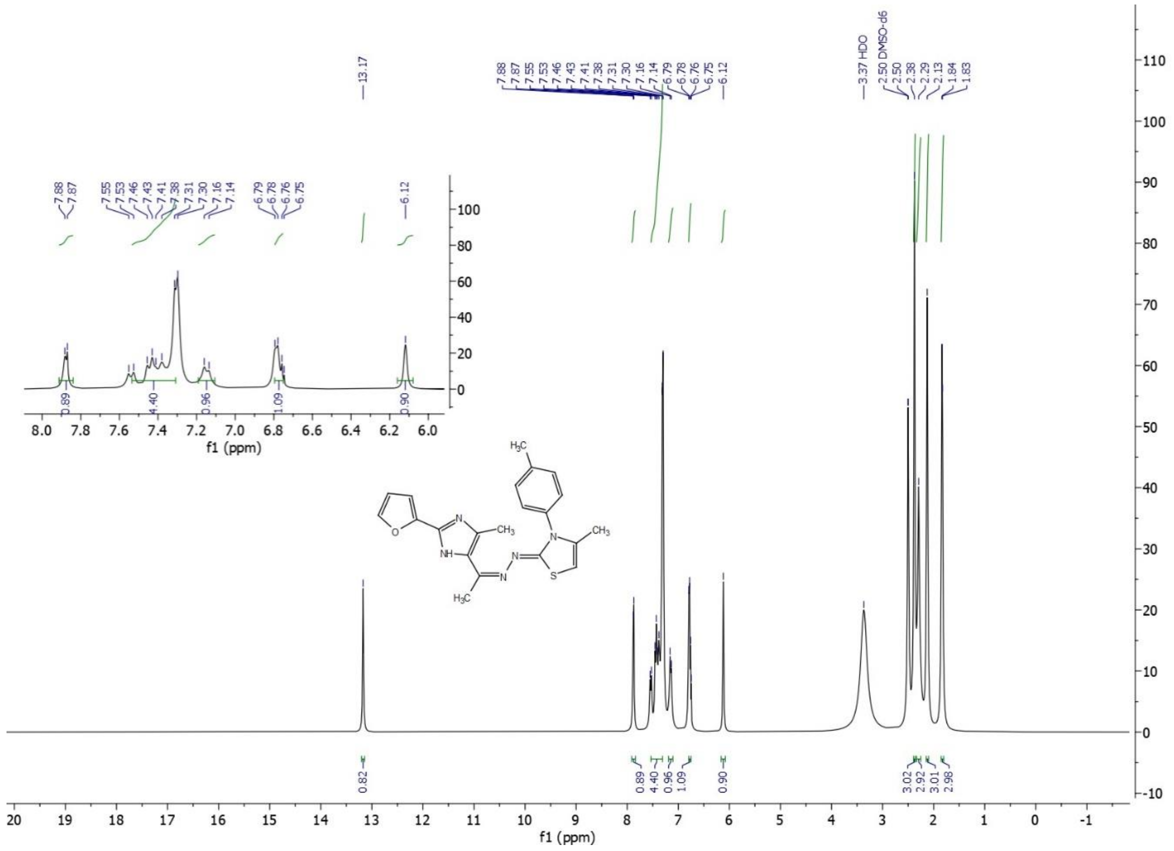

Figure S17. ^{1}H NMR Spectrum of compound **8a**

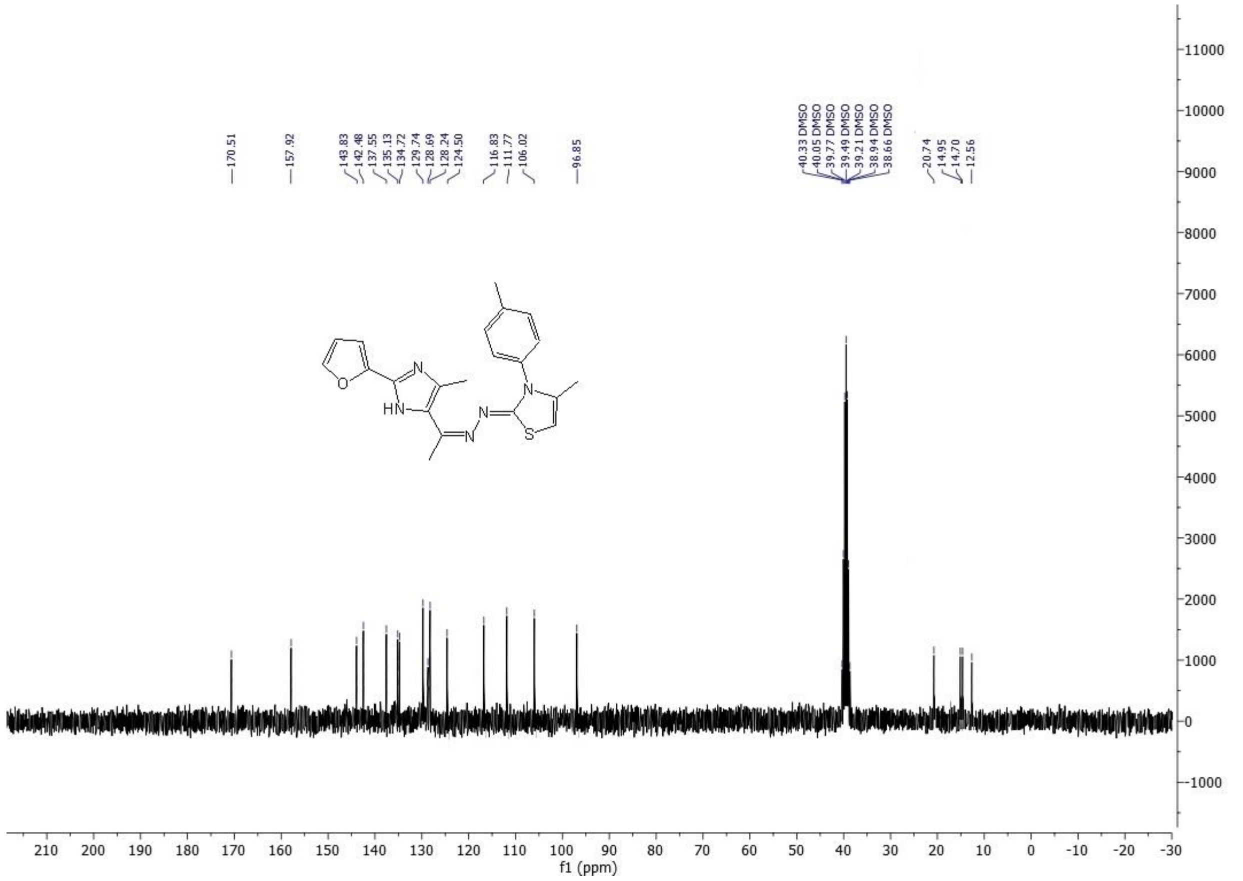

Figure S18. ^{13}C NMR Spectrum of compound **8a**

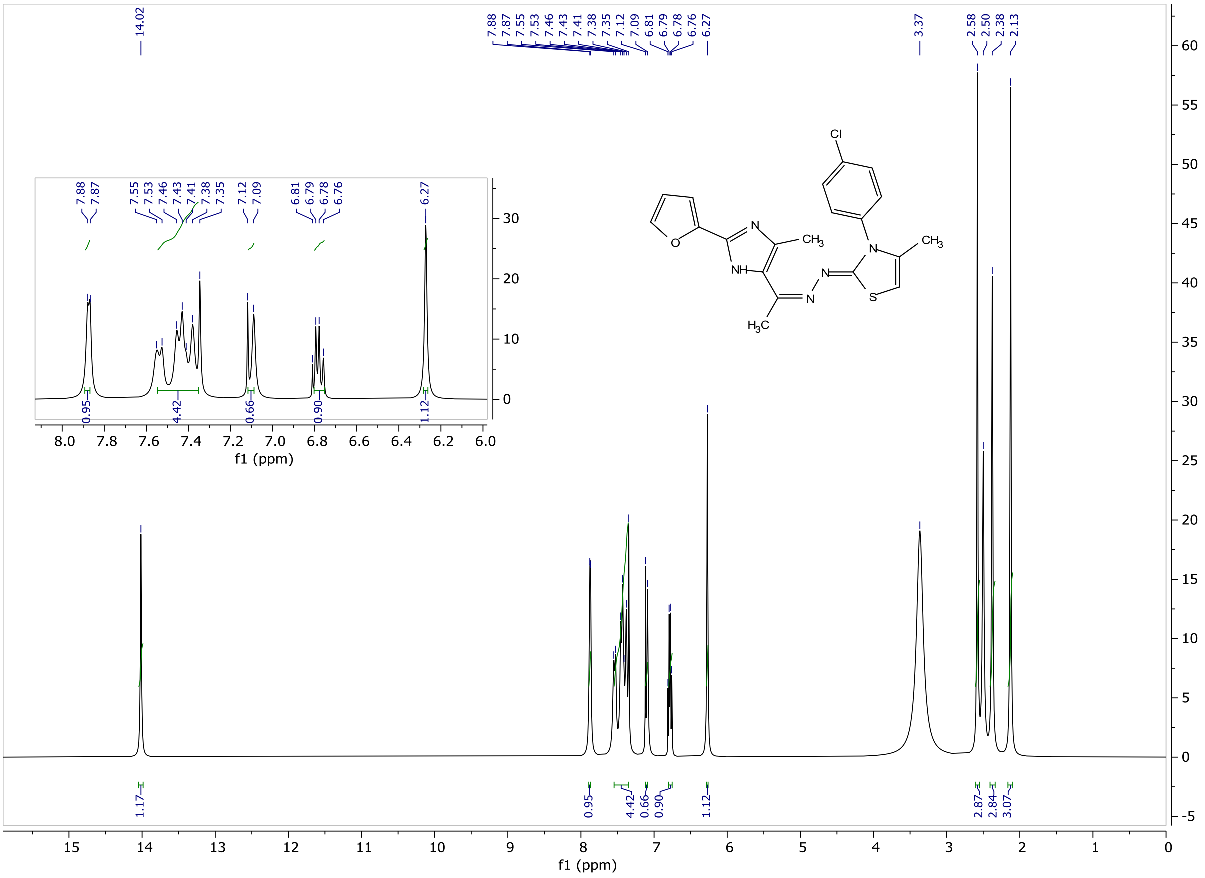

Figure S19. ^{1}H NMR Spectrum of compound **8b**

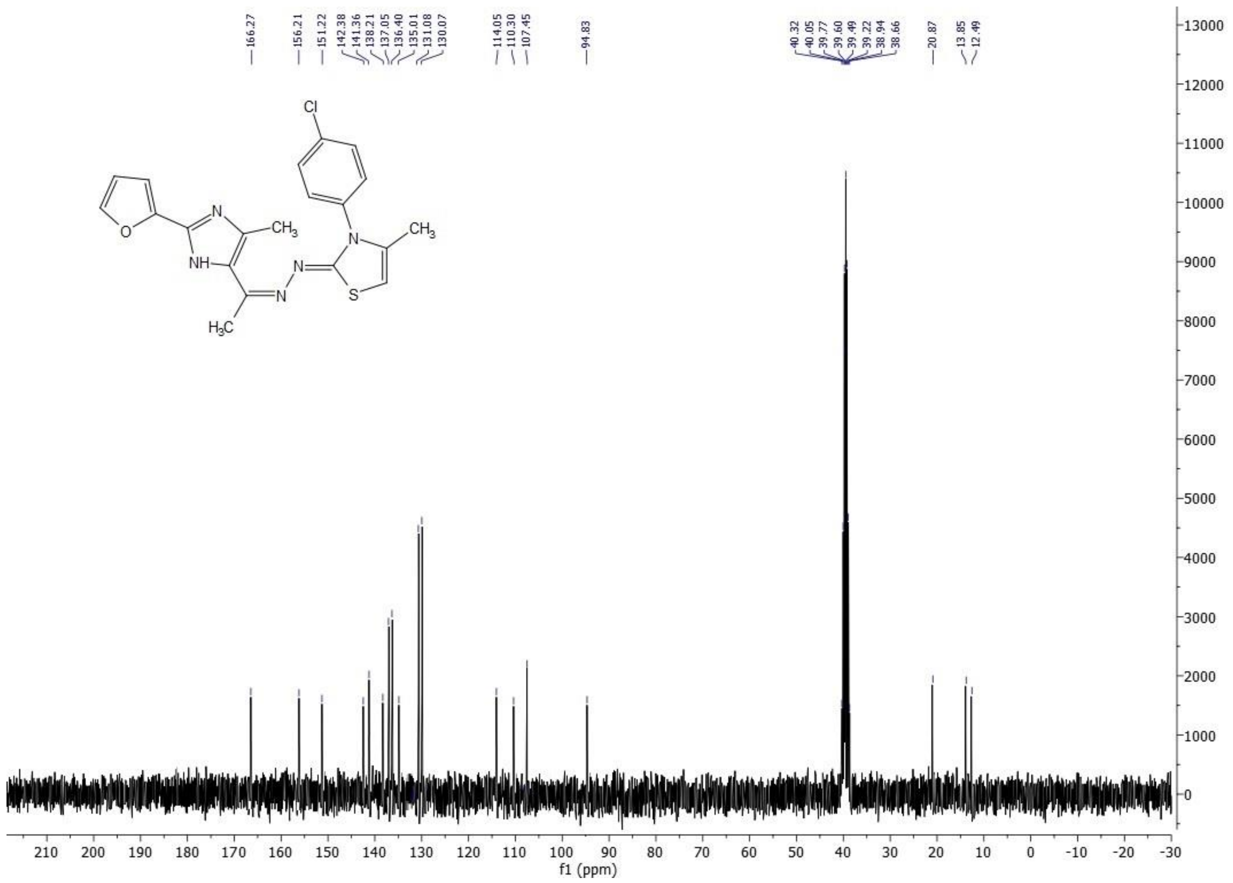

Figure S20. ^{13}C NMR Spectrum of compound **8b**

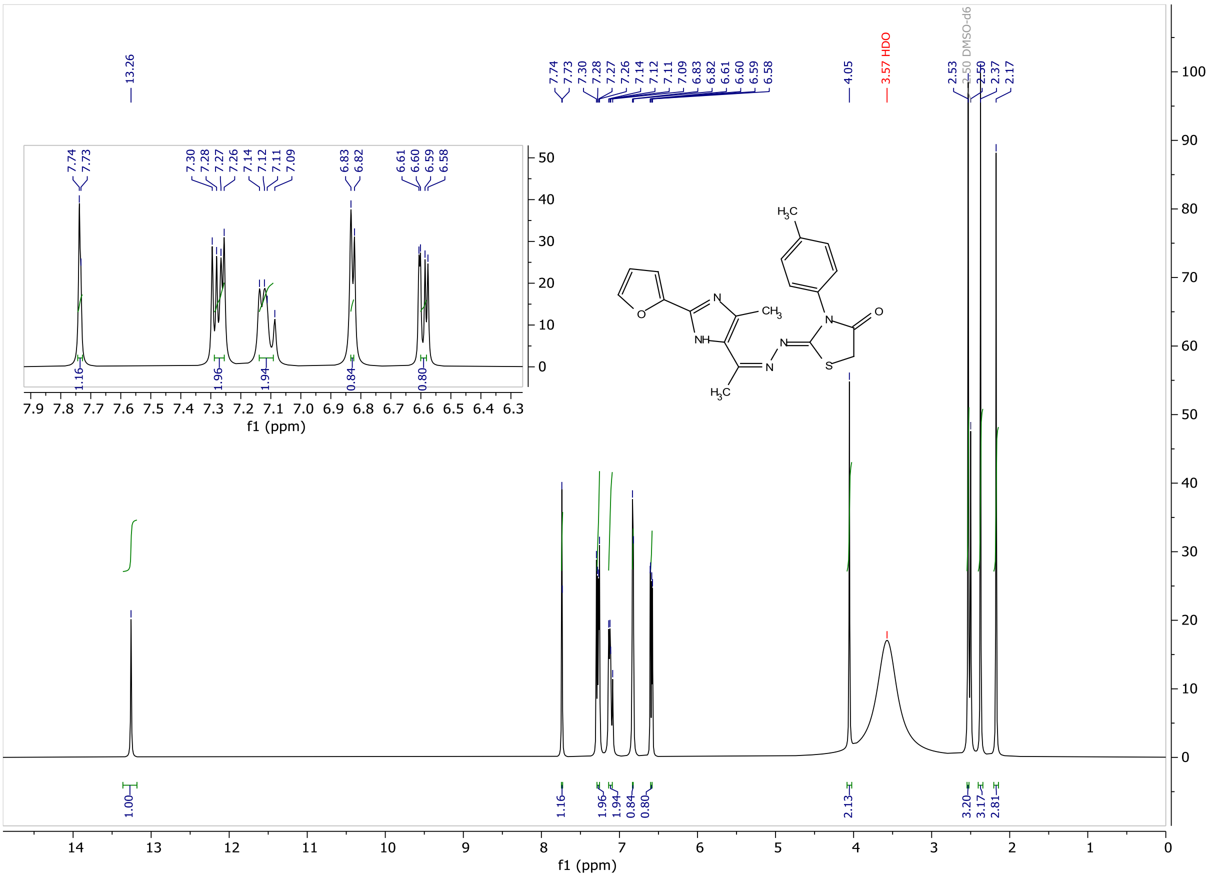

Figure S21. ^{1}H NMR Spectrum of compound **10a**

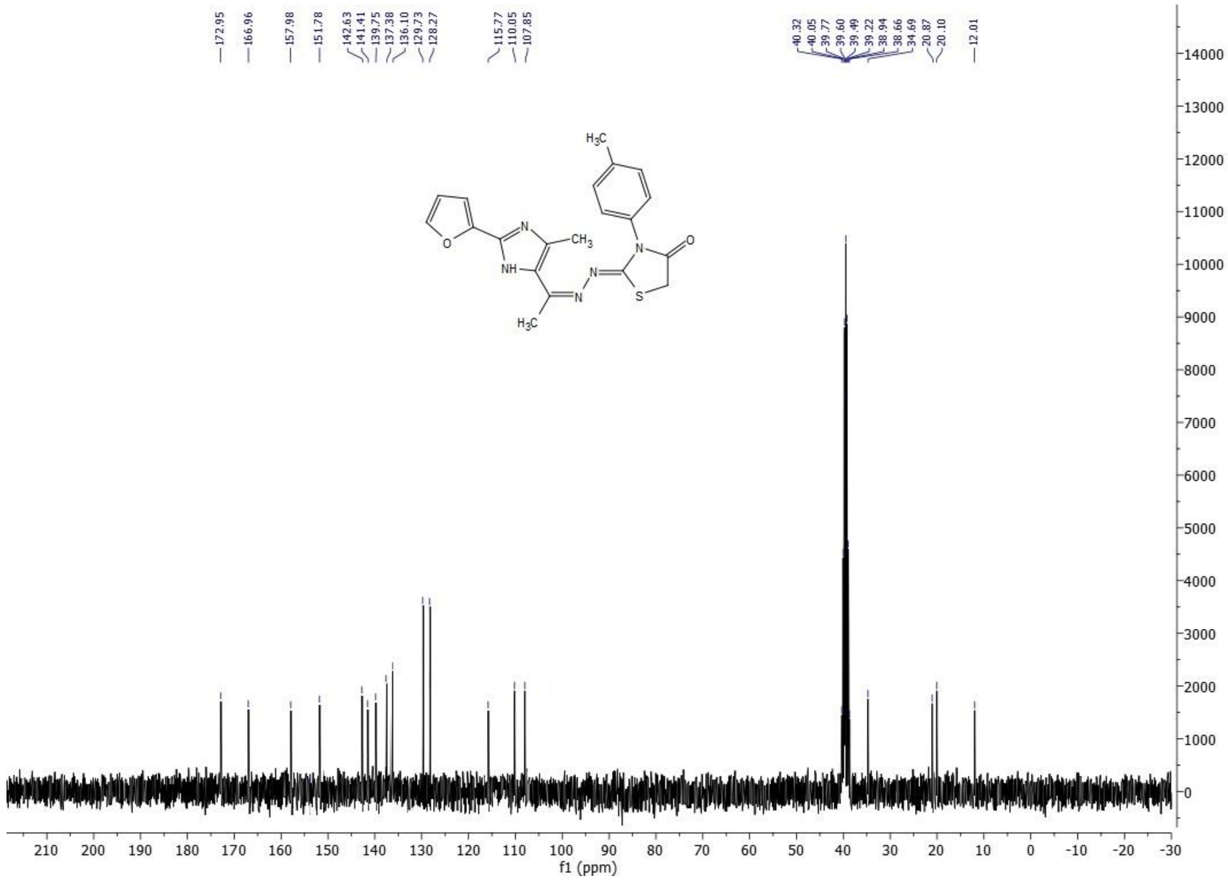

Figure S22. ^{13}C NMR Spectrum of compound **10a**

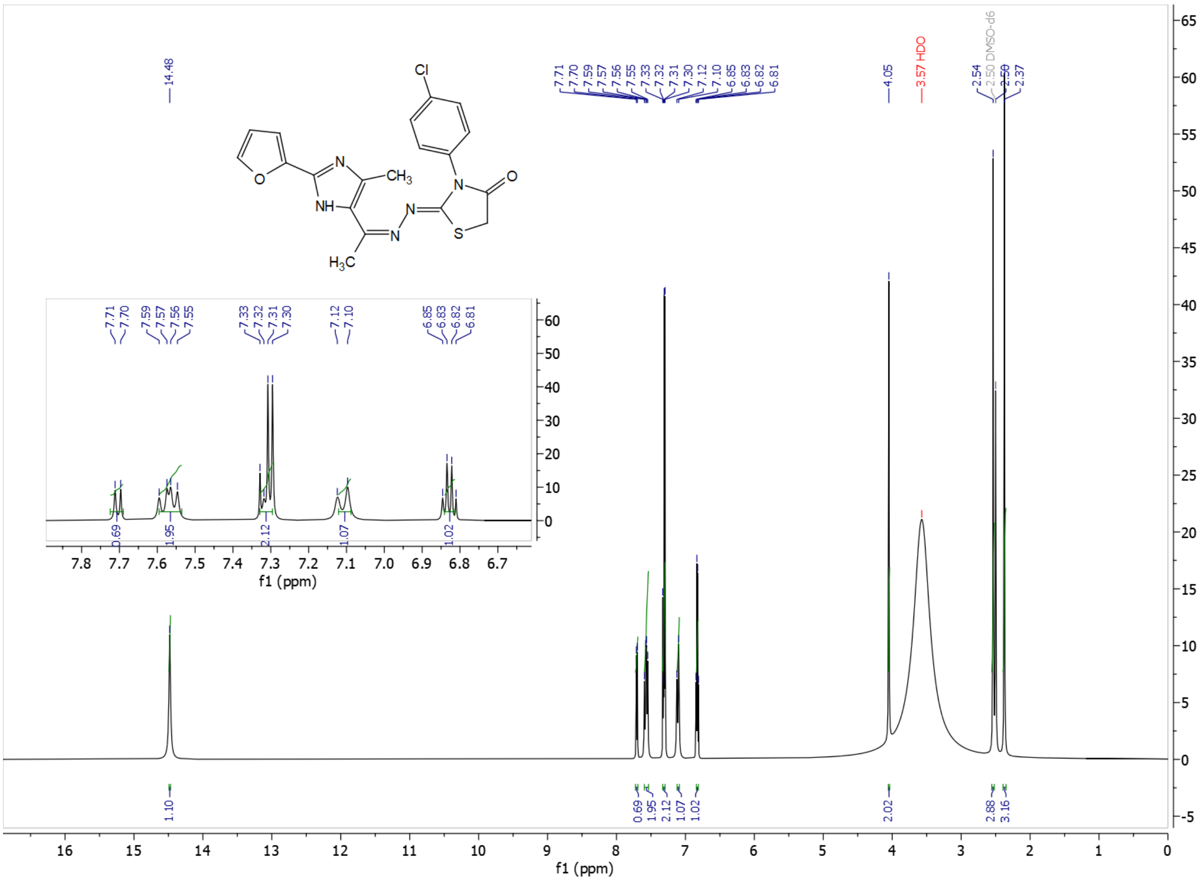

Figure S23. ^{1}H NMR Spectrum of compound **10b**

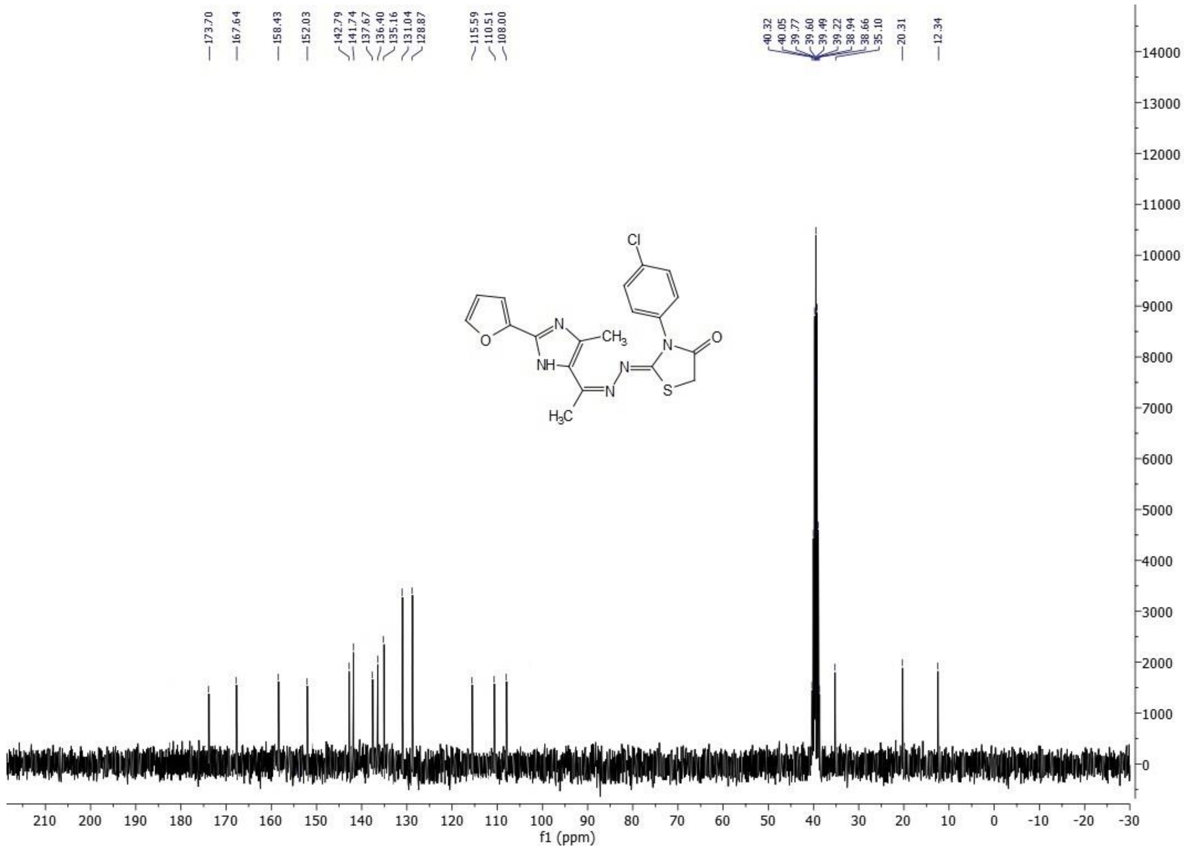

Figure S24. ^{13}C NMR Spectrum of compound **10b**

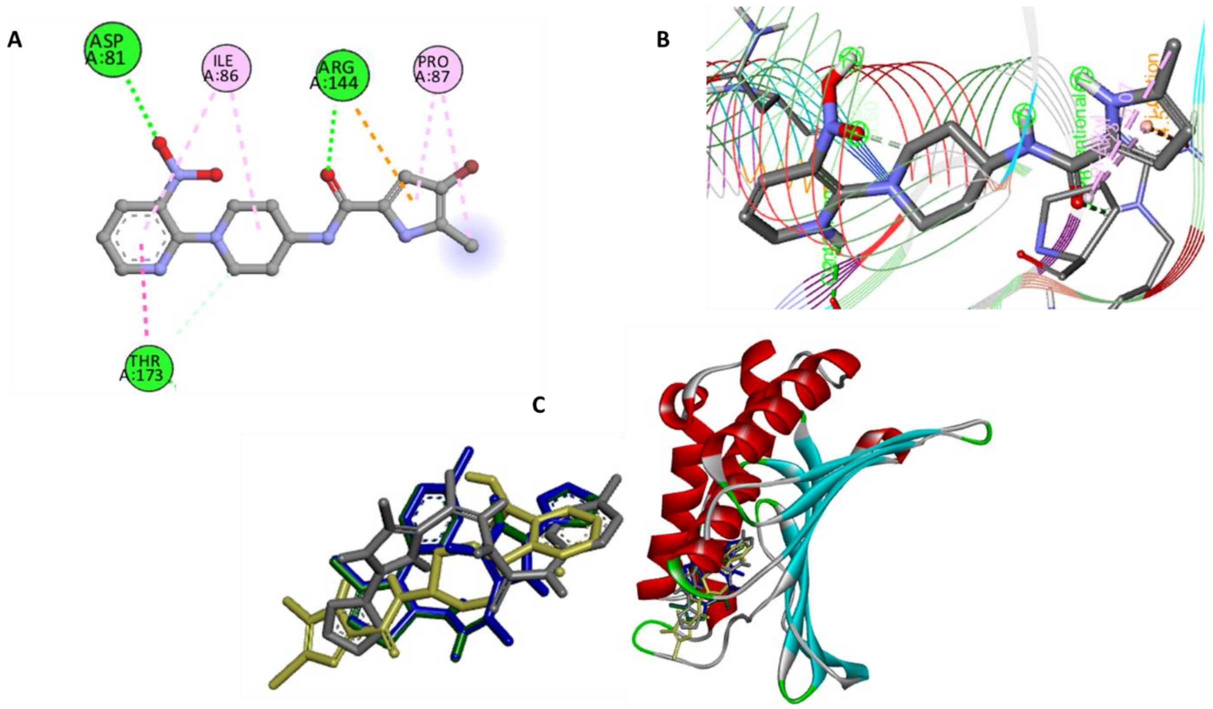

Figure S25. A: The 2D representation of the native ligand 08B inside the active pocket of *S. aureus* DNA gyrase B (PDB: 1S14). B: 3D configuration of the native ligand 08B inside the active pocket of *S. aureus* DNA gyrase B (PDB: 1S14). C: Sliding of the native ligand 08B (yellow) together compounds 3a (green), 8a (gray), and 8b (blue)

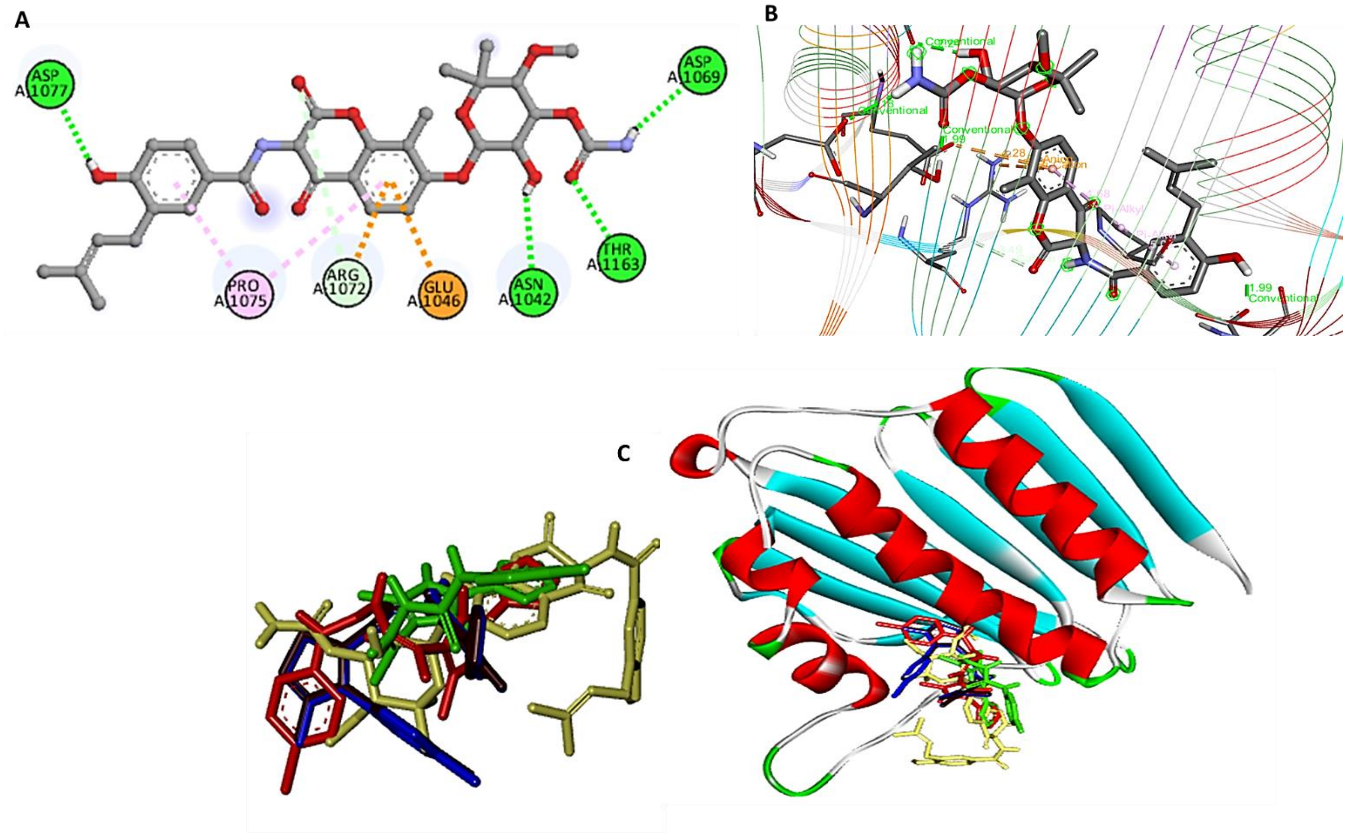

Figure S26. A: The 2D representation of the native ligand NOV inside the active pocket of E. coli DNA gyrase B (PDB: 1S14). B: 3D configuration of the native ligand NOV inside the active pocket of E. coli DNA gyrase B (PDB: 1S14). C: Sliding of the native ligand NOV (yellow) together compounds 3a (green), 3b (red), 8a (maroon), 8b (blue), and 10b (blue sky)
